# Wide‐Bandgap Organic–Inorganic Lead Halide Perovskite Solar Cells

**DOI:** 10.1002/advs.202105085

**Published:** 2022-03-08

**Authors:** Yao Tong, Adel Najar, Le Wang, Lu Liu, Minyong Du, Jing Yang, Jianxun Li, Kai Wang, Shengzhong (Frank) Liu

**Affiliations:** ^1^ Faculty of Light Industry and Chemical Engineering Dalian Polytechnic University Dalian Liaoning 116034 China; ^2^ Department of Physics College of Science United Arab Emirates University Al Ain 15505 United Arab Emirates; ^3^ Dalian National Laboratory for Clean Energy iChEM Dalian Institute of Chemical Physics Chinese Academy of Sciences Dalian Liaoning 116023 China; ^4^ Key Laboratory of Applied Surface and Colloid Chemistry Ministry of Education Shaanxi Engineering Lab for Advanced Energy Technology School of Materials Science and Engineering Shaanxi Normal University Xi'an Shaanxi 710119 China

**Keywords:** indoor photovoltaics, semitransparent perovskite solar cells, tandem solar cells, voltage loss, wide‐bandgap perovskite

## Abstract

Under the groundswell of calls for the industrialization of perovskite solar cells (PSCs), wide‐bandgap (>1.7 eV) mixed halide perovskites are equally or more appealing in comparison with typical bandgap perovskites when the former's various potential applications are taken into account. In this review, the progress of wide‐bandgap organic–inorganic hybrid PSCs—concentrating on the compositional space, optimization strategies, and device performance—are summarized and the issues of phase segregation and voltage loss are assessed. Then, the diverse applications of wide‐bandgap PSCs in semitransparent devices, indoor photovoltaics, and various multijunction tandem devices are discussed and their challenges and perspectives are evaluated. Finally, the authors conclude with an outlook for the future development of wide‐bandgap PSCs.

## Graphical abstract

In this review, the progress of wide‐bandgap organic–inorganic hybrid perovskite solar cells (PSCs) are initially summarized and the issues of phase segregation and voltage loss are assessed. Then, the diverse applications of wide‐bandgap PSCs in semitransparent devices, indoor photovoltaics, and tandem devices are discussed and their challenges and perspectives are evaluated.

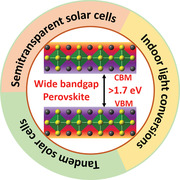

## 1 Introduction

State‐of‐the‐art metal halide perovskite materials have sparked extensive research attention due to their high absorption coefficient from their direct bandgap (*E*_g_),^[^ ^1^, ^2^ ^]^ long diffusion length of charge carriers,^[^ ^3^, ^4^ ^]^ and simple low‐temperature solution processability.^[^ ^5^, ^6^ ^]^ The meteoric rise of perovskite solar cells (PSCs) has placed them as a potential rival to compete with commercialized photovoltaics such as CdTe and silicon solar cells since the PSC power conversion efficiency (PCE) has skyrocketed from 3.8% to 25.7% in the past decade.^[^ ^7^ ^]^ The general chemical formula of three‐dimensional (3D) metal halide perovskites is ABX_3_, where A is a monovalent cation such as Cs^+^, formamidine (FA^+^), or methylammonium (MA^+^), B is a divalent metal cation involving Sn^2+^ or Pb^2+^, and X is a monovalent halide anion such as Br^−^ or I^−^. Density functional theory calculations for perovskites demonstrate that the B‐p orbitals dominate the conduction band and the valence band is composed of B‐s and X‐p orbitals.^[^ ^8^ ^]^ A cation does not contribute to the energy band, while the cation size can affect *E*_g_ by influencing the lattice size. Therefore, their combination can seamlessly tune the *E*_g_ of perovskites from 1.24 eV, such as for FA_0.75_Cs_0.25_Sn_0.5_Pb_0.5_I_3_, to 2.4 eV, such as for CsPbBr_3_. We took the liberty of dividing the whole *E*_g_ range into three parts: narrow bandgap (NBG) from 1.2 to 1.4 eV,^[^ ^9^ ^]^ typical bandgap (TBG) from 1.4 to 1.7 eV and wide bandgap (WBG) from 1.7 to 2.4 eV. This facile tunability renders them appealing in many fields of application. The NBG perovskites are beneficial not only for single‐junction PSCs accessing the Shockley–Queisser (SQ) efficiency limit of ≈33.7% and acting as bottom absorbers in tandem photovoltaics but also for the construction of a wide‐range of photodetectors and field‐effect transistors.^[^ ^10^, ^11^ ^]^ The TBG perovskites have conferred PSCs with outstanding advancements, which have been summarized from various perspectives.^[^ ^12^, ^13^, ^14^, ^15^ ^]^ The WBG perovskites are of significance in semitransparent devices, dim indoor light conversion, photoelectrochemistry conversions generating energy‐storing chemicals, tandem devices, spectrum‐splitting systems, and light emitting diodes.

The WBG perovskites can be 3D organic–inorganic hybrid‐metal mixed‐halide perovskites, (quasi) two‐dimensional (2D) perovskites, inorganic perovskites, and perovskite quantum dots. Among them, (quasi) 2D perovskites with excellent humidity resistance exhibit undesired photoelectric properties since it is an arduous task to obtain a pure (quasi) 2D perovskite structure with the vertically grown phase.^[^ ^16^ ^]^ Inorganic perovskites withstand high thermal‐decomposition temperatures,^[^ ^17^ ^]^ but this also implies high‐temperature processing is essential, even for the inorganic‐rich hybrid perovskites such as FA_0.15_Cs_0.85_PbI_3_,^[^ ^18^ ^]^ thus limiting their compatibility. Moreover, the small Goldschmidt tolerance factor induced by the small Cs^+^ leads to easy phase transition, and the transition kinetics can be hastened by atmospheric moisture or organic vapors.^[^ ^19^ ^]^ The development of perovskite quantum dots in solar cells is in its infancy, as evidenced by the low device efficiency.^[^ ^20^, ^21^ ^]^

In comparison with other WBG perovskites, organic–inorganic hybrid lead mixed‐halide perovskites are preferred and WBG perovskite compositions have been fully developed. In this article, we systematically summarize the development of WBG organic lead halide perovskites by focusing on the material composition, optimization strategy, and device performance, as well as the issues of phase segregation and voltage loss. Then we systematically discuss the diverse applications of WBG PSCs in semitransparent devices, indoor light conversion, and multi‐junction tandem devices. This review provides guidance for enhancing the performance of WBG PSCs and deepens the researcher's understanding of their application fields.

## 2 WBG Organic Lead Mixed‐Halide Perovskites

### 2.1 Organic Lead Bromide Perovskites

Both MAPbBr_3_ and FAPbBr_3_ crystallize in the pseudocubic perovskite structure Pm3̅m, and the lattice parameters for MAPbBr_3_ and FAPbBr_3_ are 5.92 and 5.99 Å, respectively.^[^ ^22^ ^]^ MAPbBr_3,_ the first reported WBG perovskite material, was applied as a sensitizer for photovoltaic cells.^[^ ^23^ ^]^ Compared to MAPbI_3_, MAPbBr_3_ exhibits much better phase stability toward both heat and moisture because of its closely packed cubic crystal lattice and the stronger Pb—Br bond. Along with the rapid development of MAPbI_3_, MAPbBr_3_‐based PSCs were also developed since its photovoltage has great potential to reach 1.9 V, although their theoretical maximum current density is 12.5 mA cm^−2^ under standard test conditions (STC, AM1.5G, 100 mW cm^−2^) due to the absorption threshold of 550 nm. In earlier studies, the optimization of MAPbBr_3_‐based PSCs was dominantly focused on judiciously selecting carrier transport layers (CTLs) to achieve suitable band alignment since, at that time, the energy loss, or the open‐circuit voltage (*V*_OC_) loss (defined as *E*_g_ − q*V*_OC_), was considered to be triggered by band mismatch.^[^ ^24^, ^25^ ^]^ A series of novel hole transport layers (HTLs)^[^ ^26^, ^27^, ^28^, ^29^, ^30^, ^31^ ^]^ with deep highest occupied molecular orbital and electron transport layers (ETLs)^[^ ^32^ ^]^ with high lowest unoccupied molecular orbital were utilized. To date, the champion PCE of MAPbBr_3_‐based PSCs is 10.4% for a device with PIF8‐TAA in combination with HBr as additives in perovskites.^[^ ^31^ ^]^ In particular, Hu et al. simultaneously modified the anode and cathode interfaces where high‐work‐function MoO*_x_* can minimize the energy barrier height and ZrO_2_ can block holes effectively, leading to restrained carrier recombination and large quasi‐Fermi level splitting (QFLS). As a result, a record *V*_OC_ of 1.65 V with a high PCE of 10.08% was obtained for MAPbBr_3_ devices.^[^ ^33^ ^]^

Liang et al. exhibited that the severe non‐radiative recombination in MAPbBr_3_ is actually a crucial factor affecting the performance of PSCs since they attained a high PCE of 8.7% and *V*_OC_ of 1.57 V from an FTO/TiO_2_/MAPbBr_3_/carbon device.^[^ ^34^ ^]^ Therefore, obtaining high‐quality films is also essential for MAPbBr_3_ devices. The crystallization of perovskite proceeds by Ostwald ripening, where the nucleation is determined by supersaturation and the crystal growth is mainly driven by ion diffusion to the as‐formed nuclei. Given that the initial nucleation of Br‐rich perovskites is accelerated by the low solubility of bromides and that the smaller ionic radius of Br^−^ (*r* = 1.96 Å) in comparison with I^−^ (*r* = 2.20 Å) leads to faster diffusivity according to the Stokes–Einstein equation, the Br‐rich perovskites tend to grow uncontrollably, resulting in incomplete coverage and pinholes. To obtain outstanding films, a range of deposition methods involving morphology optimization with Cl additives,^[^ ^27^, ^35^ ^]^ vapor‐assisted deposition,^[^ ^36^, ^37^ ^]^ anti‐solvent engineering,^[^ ^38^, ^39^ ^]^ and controlled solvent drying^[^ ^40^ ^]^ were developed, which resulted in *V*_OC_ of ≈1.4–1.5 V. For instance, Noel et al. deposited uniform, highly crystalline MAPbBr_3_ perovskite films by using acetonitrile/methylamine as solvents.^[^ ^41^ ^]^ Combining that with choline chloride passivation, they achieved MAPbBr_3_ based‐PSCs that delivered a PCE up to 8.9%, with corresponding *V*_OC_ of 1.47 V.

Although some recent results proved that MA^+^‐based perovskites possess excellent thermal stability,^[^ ^42^ ^]^ it is generally considered that they are intrinsically thermally unstable at temperatures over 85 °C even in an inert atmosphere, especially the pure MAPbI_3_ perovskite.^[^ ^43^ ^]^ Furthermore, MA^+^ possesses a high polarity (dipole of 2.29 D) compared with the lowly polar organic cation FA^+^ (dipole of 0.21 D), so the former exhibits a much stronger affinity toward moisture and polar solvents.^[^ ^44^ ^]^ Therefore, attention was shifted to FA^+^‐based perovskites. In 2014, Fabian et al. first presented highly efficient FAPbBr_3_ PSCs with a PCE approaching 7% and found FAPbBr_3_ exhibits much longer carrier diffusion length than MAPbBr_3_.^[^ ^45^ ^]^ To further enhance the PCE, lithium salt was utilized to modify the TiO_2_/FAPbBr_3_ interface, lowering the interfacial energetic disorders, thereby accelerating electron injection and lowering the charge‐carrier recombination rate. Then the FAPbBr_3_ devices yielded a marvelous *V*_OC_ of 1.53 V and a PCE above 8%. The unencapsulated FAPbBr_3_ PSCs maintained over 95% of the initial PCE after 150 consecutive hours of full‐sun illumination in nitrogen.^[^ ^46^ ^]^

### 2.2 MAPb(I_1−_ *_x_* Br*_x_*)_3_ and Phase Segregation

Substituting I^−^ with Br^−^ to yield MAPb(I_1−_ *_x_* Br*_x_*)_3_ perovskites enables tuning of *E*_g_ from 1.55 to 2.4 eV.^[^ ^2^ ^]^ Meanwhile, the tetragonal phase is preserved until *x* = 0.13 and then transforms into a cubic phase at *x* = 0.2, coupled with enhanced moisture stability. Notably, the feasible ion miscibility will also trigger easy ionic diffusion. Hence, although all MAPb(Br*_x_* I_1−_ *_x_*)_3_ perovskites have comparable Urbach energies in the range 12–17 meV,^[^ ^47^ ^]^ severe phase segregation occurs in both thin films and single crystals under either constant illumination (even less than 0.05 sun), current injection,^[^ ^48^ ^]^ or even inert, dark conditions,^[^ ^47^ ^]^ triggered by the emergence of a lower‐*E*_g_ I‐rich minority that surrounds the higher‐*E*_g_ Br‐enriched majority domains. The I‐rich minority is limited by the ultimate composition of MAPb(I_0.8_Br_0.2_)_3_ (*E*_g_ = 1.68 eV), where a cubic‐to‐tetragonal phase transition barrier prevents further segregation.^[^ ^49^ ^]^

It appears that the phase segregation is influenced by the photogenerated carrier concentration and halide defects.^[^ ^49^, ^50^ ^]^ Apart from these inherent properties, the magnitude of phase segregation depends on the operating conditions, for example, single perovskite films or complete cells. Phase segregation is negligible under short‐circuit conditions and even under maximum power point conditions compared to that under open‐circuit conditions.^[^ ^51^ ^]^ As for the driving force of phase segregation, Brivio et al. demonstrated the thermodynamic miscibility gap in MAPb(I_1−_ *_x_* Br*_x_*)_3_ below 343 K caused by lattice strain and I/Br ionic size mismatch, leading to the tendency for spontaneous order resulting in phase segregation.^[^ ^52^ ^]^ Considering the giant photostriction effect in MAPbI_3_ perovskites,^[^ ^53^ ^]^ Yang et al. deduced that the thermodynamic miscibility gap combined with the prominent photostriction results in a direct relation between the internal bonding environment and phase segregation.^[^ ^54^ ^]^ To date, the prevailing belief is that the higher concentration of photogenerated carriers can induce localized lattice strain via electron‐phonon coupling, which can easily lead to the nucleation of a light‐stabilized I‐rich minority along the grain boundaries since the perovskite lattice is ionic and soft.^[^ ^55^, ^56^, ^57^ ^]^

The phase segregation is fully reversible and thermodynamically repeatable.^[^ ^58^ ^]^ Regarding the dynamics, it takes place rapidly at a rate constant of 0.1–0.3 s^−1^ (405 nm laser, 25 mW cm^−2^ to 1.7 W cm^−2^), but the recovery driven by the entropy effect takes a few minutes to a few hours.^[^ ^59^ ^]^ More seriously, the recovery of the external quantum efficiency (EQE) for the device in the dark is much slower than that of film absorption.^[^ ^60^ ^]^ Further studies focused on suppressing the phase segregation in order to increase the PCE/stability for MAPb(I_1−_ *_x_* Br*_x_*)_3_ PSCs. Since the phase segregation takes place via halogen vacancies existing at grain boundaries, it can be mitigated in the perovskites with increased grain size and vertically oriented grain boundaries, which can be achieved by various developing deposition methods (e.g., halide exchange,^[^ ^61^ ^]^ vacuum‐deposition,^[^ ^62^ ^]^ and sequential confined‐space sublimation^[^ ^63^ ^]^) and by optimizing the crystallization process (e.g., solvent annealing, crystallization on non‐wetting substrates, and antisolvent bath).^[^ ^64^, ^65^ ^]^ For instance, Gu et al. proposed a confined‐space sublimation method which exposed solution‐processed PbX_2_ (X is I, Br, and Cl) films to MAI vapor, and the displacement of multiple halogens in a gas−solid reaction led to large grains, which endowed PSCs (*E*_g_ = 1.71 eV) with a maximum PCE of 17.9%.^[^ ^63^ ^]^ Meanwhile, surface passivation with various fullerene derivatives (e.g., [6,6]‐phenyl‐C_61_‐butyric acid methyl ester (PCBM) and indene‐C_60_ tri‐adducts) and an electron‐donating ligand (e.g., trioctylphosphine oxide (TOPO)) can also reduce nonradiative recombination and considerably restrain phase segregation.^[^ ^66^, ^67^ ^]^ Khadka et al. utilized long alkyl chain‐substituted fullerene derivatives of C_60_‐fused N‐methylpyrrolidine‐meta‐dodecyl phenyl (C_60_MC_12_) as ETLs for PSCs based on MAPbI_2.2_Br_0.8_ (*E*_g_ = 1.71 eV).^[^ ^68^ ^]^ The devices displayed an increased PCE of 16.7% with *V*_OC_ of 1.24 V.

As mentioned in Section 2.1, the low solubility and fast diffusion capability of Br^−^ lead to rapid nucleation and uncontrollable growth in MAPbBr_3_ perovskites. In MAPb(I_1−_ *_x_* Br*_x_*)_3_ perovskites, the halide composition of the as‐prepared (before annealing) perovskite films is preferentially dominated by Br^−^. Further thermal energy is required to insert I^−^ to achieve the composition homogeneity and stoichiometric balance. Xie et al. found that mixing engineering of the A‐site cation could increase the compositional homogeneity since it can cause lattice expansion or contraction. Using small Cs^+^ can tilt the PbX_6_ octahedra and broaden *E*_g_, thus reducing the reliance of the WBG on Br^−^ concentration, meanwhile controlling the nucleation rate to restrain the Br‐induced pinholes in films and increase photostability.^[^ ^69^ ^]^ Additionally, incorporating a few large FA^+^ in the precursors can effectively accelerate the I^−^ coordination in the perovskite lattice during the spin coating and improve the composition homogeneity of the initial small crystal grains, thus achieving uniform and high‐crystallinity perovskites of high Br^−^ concentration. In this manner, such MA_0.9_FA_0.1_Pb(I_0.6_Br_0.4_)_3_ perovskites (*E*_g_ = 1.81 eV) imparted an encouraging PCE of 17.1% and *V*_OC_ of 1.21 V to the PSCs.^[^ ^70^ ^]^

### 2.3 FA‐Enriched WBG Perovskites

**Figure 1:**
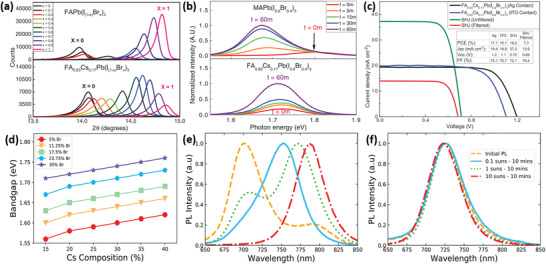
a) X‐ray diffraction results of FAPb(I_1−_ *_x_* Br*_x_*)_3_ and FA_0.83_Cs_0.17_Pb(I_1−_ *_x_* Br*_x_*)_3_; b) normalized PL measured after light exposure of the MAPb(I_0.6_Br_0.4_)_3_ and FA_0.83_Cs_0.17_Pb(I_0.6_Br_0.4_)_3_ thin films; c) *J–V* curves for the PSCs. Reproduced with permission.^[^ ^74^ ^]^ Copyright 2016, American Association for the Advancement of Science. d) *E*_g_ versus perovskite composition for Cs*_x_* FA_1−_ *_x_* Pb(Br*_y_* I_1−_ *_y_*)_3_; PL data comparing e) FA_0.83_Cs_0.17_Pb(I_0.6_Br_0.4_)_3_ and f) FA_0.6_Cs_0.4_Pb(I_0.7_Br_0.3_)_3_ perovskites. Reproduced with permission.^[^ ^75^ ^]^ Copyright 2018, American Chemical Society.

As mentioned, FA^+^‐based perovskites possess better stability than their MA^+^‐based counterparts. FAPbI*_x_* Br_3−_ *_x_* perovskites also show a wide range of *E*_g_ tunability from 1.4 to 2.23 eV. Note that the perovskite phase for *x* = 0.5, 0.6, and 0.7 is amorphous because the crystal structure transitions from cubic (for *x* < 0.5, Br‐rich) to tetragonal (*x* > 0.7, I‐rich).^[^ ^71^ ^]^ Strikingly, unlike MAPbI*_x_* Br_3−_ *_x_*, which undergoes phase segregation under weak illumination of 15 mW cm^−2^ (457 nm), FAPbI*_x_* Br_3−_ *_x_* is stable under STC because the initiating intensity is higher than 7.2 W cm^−2^ (400 nm).^[^ ^72^ ^]^ Further introducing Cs^+^ into the FAPbI*_x_* Br_3−_ *_x_* can achieve crystalline films throughout the entire compositional range by removing the structural phase transition (**Figure** **1**a), where Cs^+^ content between 0.10 < *y* < 0.30 confers FA_1−_ *_y_* Cs*_y_* PbI*_x_* Br_3−_ *_x_* perovskites with high crystalline quality, prominent carrier mobilities, and prolonged carrier lifetimes.^[^ ^73^ ^]^ Meanwhile, compared with FAPbI*_x_* Br_3−_ *_x_*, the photostability and the thermal stability are also enhanced by impeding humidity‐induced decomposition and inherent strain‐induced phase segregation (Figure 1b). FA_0.83_Cs_0.17_Pb(I_0.6_Br_0.4_)_3_ (*E*_g_ = 1.74 eV) exhibits low levels of electronic disorder with a small Urbach energy of 16.5 meV and excellent carrier mobility of 21 cm^2^ V^−1^ s^−1^. As such, PSCs based on FA_0.83_Cs_0.17_Pb(I_0.6_Br_0.4_)_3_ yielded a short‐circuit current density (*J*_SC_) of 19.4 mA cm^−2^, a *V*_OC_ of 1.2 V, and a PCE of 17.1% (Figure 1c).^[^ ^74^ ^]^ For the FA_1−_ *_y_* Cs*_y_* PbI*_x_* Br_3−_ *_x_* system, as per (Figure 1d), introducing either Cs^+^ or Br^−^ can widen the *E*_g_ by 2.3 ± 0.2 and 5.7 ± 0.2 meV/%, respectively,^[^ ^75^ ^]^ whereas for a given *E*_g_, using more Cs^+^ rather than more Br^−^ is more ideal because higher Br^−^ content will increase either the density or the depth of nonradiative recombination sites and decrease thermodynamic favorability in theory.^[^ ^76^ ^]^

For instance, FA_0.83_Cs_0.17_PbI_0.6_Br_0.4_ and FA_0.6_Cs_0.4_PbI_0.7_Br_0.3_ possess an identical *E*_g_ of 1.75 eV, but the PSCs based on the latter yield higher PCE, increased *V*_OC_ and improved photostability (Figure 1e,f).^[^ ^75^ ^]^ Certainly, excessive Cs^+^ content will accelerate the perovskite degradation possibly caused by the insufficient crystallinity. It is notable that Cs^+^ plays a paramount but inconclusive role in increasing photostability for the mixed‐cation perovskite system since Chen et al. found that there exists evident photoinduced phase segregation in MA_0.6_FA_0.4_Pb(I_0.6_Br_0.4_)_3_ and FA_0.6_MA_0.4_Pb(I_0.6_Br_0.4_)_3_ films, while it is negligible in Cs_0.6_MA_0.4_Pb(I_0.6_Br_0.4_)_3_ and Cs_0.6_FA_0.4_Pb(I_0.6_Br_0.4_)_3_.^[^ ^77^ ^]^

Thanks to the above advancements, the FA_1−_ *_y_* Cs*_y_* PbI*_x_* Br_3−_ *_x_* system started to flourish among the WBG PSCs. Note that although FA_1−_ *_y_* Cs*_y_* Pb(I_1−_ *_x_* Br*_x_*)_3_ exhibited decent photostability, phase segregation accompanied by decomposition will still occur when excess bromine content (*x* > 0.6) is incorporated or under light‐soaking at elevated temperature.^[^ ^78^, ^79^, ^80^ ^]^ Additionally, the uncontrollable crystallization process triggered by the abundant yet insoluble Cs^+^/Br^−^ salts in the precursors generally leads to small perovskite grain size and significant hysteresis in devices. Thus, several optimizing strategies, which can be summarized as additive engineering, surface engineering, and mixed‐cation engineering, emerged to address the above issues and further reduced *V*_OC_ loss and increased PCE.

#### 2.3.1 Additive Engineering

Additive engineering focuses on modulating the crystallization process and passivating defects for WBG perovskites. Yan's group started to optimize FA_0.8_Cs_0.2_Pb(I_0.7_Br_0.3_)_3_ perovskite (*E*_g_ = 1.75 eV) by converging Pb(SCN)_2_ additive and the solvent‐atmosphere annealing strategy.^[^ ^81^, ^82^ ^]^ Consequently, the average grain size of perovskite layers rose from 66 ± 24 to 1036 ± 111 nm, and the carrier lifetime increased from 330 to above 1000 ns. As a result, the PSC achieved a PCE of 18.3% with a *V*_OC_ of 1.25 V. In a similar vein, Zhou et al. combined Pb(SCN)_2_ additive with a matched ratio of FAX (X = I and Br) to realize large grains coupled with defect‐healed grain boundaries.^[^ ^83^ ^]^ Notably, the perovskites exhibit excellent photostability at both room and elevated temperature. The corresponding PSCs show outstanding *V*_OC_/stabilized PCE reaching 1.24 V/18.6%, 1.28 V/16.5%, 1.30 V/15.0%, and 1.31 V/14.4% for FA_0.83_Cs_0.17_PbI_3−_ *_x_* Br*_x_* with *x* = 0.8, 1.2, 1.5, and 1.8, respectively, and corresponding *E*_g_ of 1.72–1.93 eV.

As mentioned, the high content of Cs^+^/Br^−^ in FA_0.83_Cs_0.17_Pb(I_0.6_Br_0.4_)_3_ perovskites may lead to their uncontrollable precipitation due to the low solubility, thus inducing undesired non‐active phases and defects in the perovskite films. Kim et al. utilized highly polar formamide as additive to increase the solubilization of Cs^+^ salt and allow the perovskite to directly crystallize into the black *α* phase, bypassing the undesired yellow *δ* phase.^[^ ^84^ ^]^ Consequently, the PSCs exhibited a PCE of 17.8% with a high *V*_OC_ of 1.23 V and low hysteresis. In addition, K^+^ was demonstrated as an excellent additive for FA^+^‐rich WBG perovskites.^[^ ^80^, ^85^ ^]^ K^+^ decorates the grain boundaries rather than being doped into the perovskite lattice, which can substantially mitigate both non‐radiative recombination losses and photoinduced ion migration in perovskite films and interfaces. Liang et al. deployed K^+^ in FA_0.8_Cs_0.2_Pb(I_0.7_Br_0.3_)_3_ perovskites (*E*_g_ = 1.7 eV),^[^ ^85^ ^]^ which significantly suppressed phase segregation and reduced the voltage loss from 152 to 95 mV. Therefore, the KI‐modified WBG PSCs yielded a high V_OC_ of 1.19 V along with a PCE of 18.3%.

#### 2.3.2 Surface Engineering

**Figure 2:**
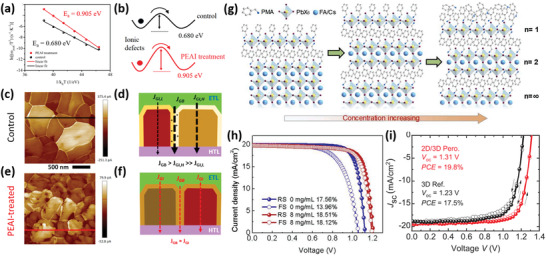
a) Arrhenius plots extracted from admittance spectra; b) schematic of the energy barrier for ion movement in control and PEAI‐treated devices. Conducting AFM results of c) control and e) PEAI‐treated perovskite films. Schematic current paths in d) the control and f) PEAI‐treated PSCs. Reproduced with permission.^[^ ^86^ ^]^ Copyright 2020, American Chemical Society. g) Schematic perovskite structure treated with different concentrations of PMABr solution. h) *J–V* curves of PSCs with and without PMABr treatment. Reproduced with permission.^[^ ^87^ ^]^ Copyright 2020, Elsevier B. V. i) *J–V* curves of PSCs with and without BABr passivation. Reproduced with permission.^[^ ^88^ ^]^ Copyright 2020, Wiley‐VCH.

Surface engineering for WBG perovskites mainly refers to deploying bulky cations on the perovskite film surface, which generally achieves defect passivation, band alignment, and formation of low‐dimension perovskites, thus further inhibiting ion migration, favoring carrier extraction and increasing resistance toward illumination/humidity. For example, Yan's group successively applied guanidinium bromide surface treatment to achieve the formation of a graded perovskite homojunction^[^ ^82^ ^]^ and phenethylammonium iodide (PEAI) treatment to increase the activation energy barrier (**Figure** **2**a,b) for ion migration and reduce the dark saturation current density.^[^ ^86^ ^]^ Especially, they pointed out that the leakage current triggers the *V*_OC_ loss in WBG PSCs through defective grain boundaries, which can be reduced by passivating surface defects through PEAI treatment (Figure 2c–f). As such, the 1.73 eV PSC delivered a PCE of 19.1% with a *V*_OC_ of 1.25 V. In particular, Zheng et al. showed that quaternary ammonium halides with negative‐ and positive‐charged components can effectively passivate ionic defects in perovskites, thus reducing the charge trap density, elongating the carrier recombination lifetime and significantly enhancing the stability of films in ambient conditions, which eventually increased the PCE of FA_0.83_MA_0.17_Pb(I_0.6_Br_0.4_)_3_ (1.72 eV) PSCs to 17.2%.^[^ ^89^ ^]^

In addition, surface‐treatment‐induced low‐dimensional perovskites can also effectively inhibit the ion migration and remove the defect sites, thus protecting perovskites from decomposition and phase segregation. Typically, Zhou et al. observed both the phase segregation and decomposition in FA_0.85_Cs_0.15_Pb(I_0.73_Br_0.27_)_3_ perovskite films under the concerted effect of heat and light, then they proposed the post‐treatment with phenylmethylamine (PMA) molecules to passivate the defective regions, thus preventing the decomposition and phase segregation. Additionally, the PMA molecules can react with the 3D perovskite to form a thin 2D PMA_2_PbI_4_. The energy cascade between 2D layer and its underneath 3D perovskite promotes hole extraction and blocks electron transport. The champion PMA‐modified FA_0.15_Cs_0.85_Pb(I_0.73_Br_0.27_)_3_ (*E*_g_ = 1.72 eV) PSCs exhibited a PCE of 18.1% and an *V*_OC_ of 1.24 V.^[^ ^78^ ^]^ Bu et al. employed phenylmethylamine bromide (PMABr) to treat the FA_0.8_Cs_0.2_Pb(I_0.7_Br_0.3_)_3_ (*E*_g_ = 1.74 eV) perovskite film surface, which formed a hierarchical layered perovskites structure of 2D perovskite layer/quasi‐2D perovskite layer/3D perovskite film (Figure 2g).^[^ ^87^ ^]^ Consequently, the PSCs with mixed 2D/3D layers reached a PCE of 18.5% with negligible hysteresis and significantly enhanced moisture resistance (Figure 2h). The post‐treatment with PMABr to improve the device performance was also confirmed by Li and co‐workers.^[^ ^90^ ^]^ Moreover, Duong et al. coated the surface of perovskite with butyl ammonium bromide (BABr) and found the formed 2D RP perovskite phase with *n* = 2 can passivate the surface defects, change the electronic structure at the 3D perovskite surface, thus resulting in prolonged carrier lifetime. With this surface strategy, they fabricated Rb_0.05_Cs_0.095_MA_0.1425_FA_0.7125_PbI_2_Br (*E*_g_ = 1.72 eV) PSCs with PCE up to 18.3%. Similarly, yet strikingly, Gharibzadeh et al. also designed a hybrid 2D/3D perovskite heterostructure by spin coating BABr onto a FA_0.83_Cs_0.17_Pb(I_0.6_Br_0.4_)_3_ (*E*_g_ = 1.72 eV) perovskite surface.^[^ ^88^ ^]^ Benefiting from the mitigated nonradiative recombination, the PSCs exhibited stable PCE of 19.4% and a remarkably high *V*_OC_ of 1.31 V (Figure 2i), exceeding 90% of the SQ limit.

In addition to the ammonium ions, Belisle et al. coated the perovskite surface with the electron‐donating ligand TOPO, which is able to both reduce nonradiative recombination and dramatically slow the onset of halide segregation in MAPbI_2_Br films.^[^ ^67^ ^]^ Besides, Jaysankar et al. successfully passivated the perovskite/HTL interface by utilizing atomic layer deposited Al_2_O_3_, which significantly enhanced the *V*_OC_ of Cs_0.15_FA_0.85_Pb(I_0.71_Br_0.29_)_3_ (*E*_g_ = 1.72 eV) PSCs to 1.22 V.^[^ ^91^ ^]^

#### 2.3.3 Mixed‐Cation Engineering

Mixed‐cation engineering can also optimize the properties of perovskites because the A‐site cations can bring in lattice strain, tilt the PbX_6_ octahedra, or form lower‐dimensional perovskite‐like structures, although it does not contribute to the energy band directly.^[^ ^92^, ^93^, ^94^ ^]^ Hillhouse et al. presented incorporating GA^+^ into the perovskite lattice while simultaneously compensating with equimolar Cs^+^ to maintain the tolerance factor. This strategy enabled the formation of a black‐phase lattice‐straining perovskite and a slightly increased *E*_g_ and achieved enhanced photostability.^[^ ^95^ ^]^ As a result, perovskite films with the compositions of (FA_0.33_GA_0.19_Cs_0.47_)Pb(I_0.66_Br_0.34_)_3_ (*E*_g_ = 1.84 eV) and (FA_0.58_GA_0.10_Cs_0.32_)Pb(I_0.73_Br_0.27_)_3_ (*E*_g_ = 1.75 eV) achieved QFLS of 1.43 and 1.35 eV, respectively, both reaching >91% of the SQ limit. Also, (FA_0.58_GA_0.10_Cs_0.32_)Pb(I_0.73_Br_0.27_)_3_ PSCs gave a *V*_OC_ of 1.24 V and a PCE of 14.2%. In addition to the GA^+^, bulky cations of acetamidinium, dimethylamine, and ethylamine also perform the function of compensating Cs^+^ and increasing *E*_g_.^[^ ^96^, ^97^ ^]^ Additionally, Tan et al. discovered a few MA^+^ in WBG perovskites can heal deep trap defects, resulting in stronger defect tolerance. PSCs based on Cs_0.12_MA_0.05_FA_0.83_Pb(I_0.6_Br_0.4_)_3_ (*E*_g_ = 1.74 eV) achieved a high stabilized PCE of 19.1% with a large *V*_OC_ of 1.25 V.^[^ ^98^ ^]^

### 2.4 Stability and Voltage Loss

Perovskite materials belong to the category of soft‐ionic‐crystal substances, and their lack of sufficient long‐term stability is a critical issue that hinders the rapid industrialization of PSCs. Not only does it limit application of perovskite in single‐junction devices, it also limits its application as an add‐on device in multijunction devices because the existing photovoltaic technologies, such as silicon solar cells and copper indium gallium diselenide (CIGS), have passed the toughest, most challenging standard testing conditions. Perovskite materials possess a significant response correlation with external conditions involving light illumination, high temperature, electrical bias, and atmospheric conditions. Discussions about improving the stability of TBG PSCs have been reported in numerous articles.^[^ ^99^, ^100^, ^101^, ^102^ ^]^ For example, the atmospheric stability can be enhanced by introducing low‐dimensional perovskites as protecting layers,^[^ ^103^ ^]^ improving polycrystalline quality, and developing package technology.^[^ ^104^ ^]^ In addition, thermal stability can be attained by introducing low‐polarity cations to replace the polar ones.^[^ ^105^ ^]^

For WBG perovskites, there is a subtle relation between the bandgap of the perovskites and the device stability of the corresponding solar cells. First, the bandgap region of 2.2–2.3 eV corresponds to the perovskites of MAPbBr_3_ and FAPbBr_3_. Due to the closely packed perovskite lattices, these Br‐based perovskites are stable against humidity/O_2_ in the ambient air, and they also possess excellent photo stability devoid of phase segregation. Compared with MAPbBr_3_, FAPbBr_3_ generally exhibit superior thermal stability and humidity stability because of the lowly polar organic cation of FA^+^. Besides, FAPbBr_3_ has a relatively stable pseudocubic structure, in contrast to FAPbI_3_ that spontaneously forms a non‐perovskite yellow *δ*‐phase.^[^ ^45^ ^]^ For example, the unsealed FAPbBr_3_ devices prepared by Arora et al. displayed unprecedented photostability of over 150 h under continuous illumination and outstanding stability upon long‐term aging (4000 h).^[^ ^46^ ^]^ Second, the bandgap of 1.9–2.2 eV requires the Br content >60% for the I/Br alloyed perovskite system. However, the miscibility limit exists for Br content higher than 40%, even in the Cs^+^ and FA^+^ containing mixed halide perovskites.^[^ ^79^ ^]^ Noting that, perovskites with bandgap in this range are rarely reported, and thus they still suffer from severe photoinstability and phase separation. Last, the perovskites with the bandgap of 1.7–1.9 eV generally employ the Br concentration lower than 50%. Since the stability issues caused by high energetic disorder and serious halide phase segregation have been addressed by McMeekin et al., who demonstrated a series of efficient FA/Cs‐based WBG PSCs,^[^ ^106^ ^]^ such perovskites have been extensively studied and most of the optimization strategies summarized above are aimed at these perovskites to improve their performance and stability. For instance, Snaith's group prepared BA_0.09_(FA_0.83_Cs_0.17_)_0.91_Pb(I_0.6_Br_0.4_)_3_ perovskite that brings significantly improved perovskite performance and enhanced operational stability in PSCs. A champion *t*_80_ lifetime of 3880 h for PCE and 1680 h for stabilized power output was achieved in the encapsulated device.^[^ ^107^ ^]^

Since the increment in the bandgap is accomplished by partially substituting the original I‐ to Br^‒^/Cl^‒^ or FA^+^/MA^+^ to Cs^+^ in the perovskite absorber, all the perovskites with bandgap between 1.7 and 2.2 eV are still not completely free from the phase separation issues, which is due to the following reasons: i) the uncontrollable crystallization process caused by the low solubility of precursors will lead to poorly crystallized WBG perovskites with more grain boundaries and surface defects; ii) the resulting lattice mismatch will trigger more severe ion migration in the mixed perovskites compared with TBG perovskites. In particular, Li et al. recently demonstrated that light‐generated carriers drive the formation of a Cs‐rich phase in FA_0.9_Cs_0.1_PbI_3_ perovskites, which leads to considerable operational efficiency loss in spite of the reasonable thermal stability.^[^ ^108^ ^]^ iii) The dynamic ion migration process can be promoted by illumination and high temperature, thus the phase segregation and performance degradation will be aggravated under the combination of illumination and high temperature, or even under International Standards (IEC 61646 climatic chamber tests) for testing; iv) the undesired band alignment between the WBG perovskites and CTLs will lead to charge accumulation. Note that both the defect states and charge accumulation can accelerate phase segregation and performance degradation. Overall, the stability of WBG perovskites is still an issue of concern.

**Figure 3:**
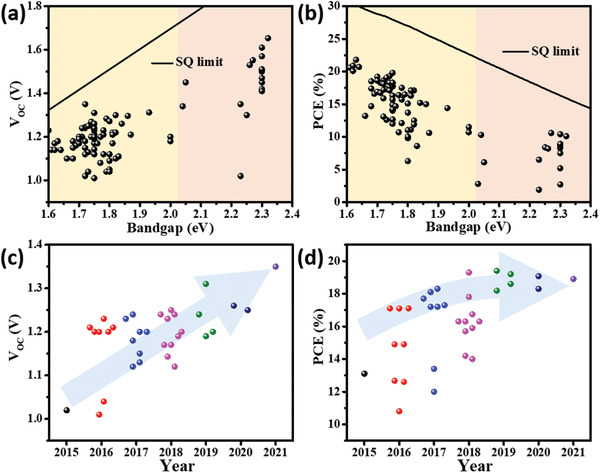
a) *V*_OC_ and b) PCE of PSCs based on WBG perovskites; c) *V*_OC_ and d) PCE of PSCs based on 1.70–1.75 eV perovskites versus time. The detailed data are listed in Table S1, Supporting Information.

The *V*_OC_ loss (defined as *E*_g_− q*V*_OC_) in the TBG PSCs ranges from 0.35 to 0.45 V, and strikingly, it has be reduced to 0.34 V for the 1.6 eV PSCs by optimizing the perovskites and contacts.^[^ ^109^ ^]^ In contrast, further increasing *E*_g_ does not impose a corresponding increment in *V*_OC_. The *V*_OC_ “‘plateau”’ at ≈1.2 V accompanied by hundreds of millivolts of *V*_OC_ loss in APb(I_1−_ *_x_* Br*_x_*)_3_ PSCs was proposed in 2018.^[^ ^110^ ^]^ **Figure** **3**a,b updates the *V*_OC_ and PCE of PSCs based on WBG perovskites. Apparently, the *V*_OC_ “‘plateau”’ has shifted to ≈1.3 V so far, implying a *V*_OC_ loss of 0.40–0.45 V for the 1.70–1.75 eV perovskites. Strikingly, Liu et al. has demonstrated *V*_OC_ of 1.35 V and PCE of 18.9% in an MAPb(I_0.8_Br_0.2_)_3_ (*E*_g_ = 1.72 eV) device by tuning the band alignment and reducing interface recombination.^[^ ^111^ ^]^ Figure 3c,d displays the development of *V*_OC_ and PCE of PSCs utilizing perovskites ranging 1.70–1.75 eV. Intriguingly, the *V*_OC_ increased, while the PCE witnessed negligible increment, constantly lower than 20%, thus obviously indicting that the *V*_OC_ increased at the cost of *J*_SC_, probably caused by the low carrier injection rate.^[^ ^112^ ^]^

Generally, apart from the unavoidable SQ loss, such a large *V*_OC_ loss in WBG PSCs arises from i) poor film quality along with optoelectronic characteristics, ii) improper interfacial energetics and mismatched energy levels at the interfaces and undesirable CTLs. For instance, Stolterfoht et al. quantified the *V*_OC_ loss contributed by the loss (135 mV) in the perovskite bulk as well as interfacial recombination loss of 80 mV at each interface.^[^ ^113^ ^]^

The film quality is a combination of crystallinity, morphology, phase stability, etc. Improper film quality implies an ocean of charged defects in perovskites, especially on the surface and at grain boundaries, that can capture carriers. Since some of the trapped charges may escape over a long time and be collected by the electrodes, they may not significantly influence the photocurrent output, while the resulting energy disorder and low carrier concentration would significantly pull down the QFLS, thus affecting the *V*_OC_. The film quantity can be denoted as *χ* = QFLS/*q* $V_{\mathrm{OC}}^{\mathrm{SQ}}$,^[^ ^110^ ^]^ where QFLS is determined by the photoluminescence quantum yield^[^ ^114^ ^]^ of the perovskite films and the $V_{\mathrm{OC}}^{\mathrm{SQ}}$ is the theoretical limit of *V*_OC_.^[^ ^115^ ^]^ The prevailing reason behind the large *V*_OC_ loss is the phase segregation in perovskites. However, recent results argued that phase segregation is not the dominant mechanism for *V*_OC_ loss in WBG PSCs.^[^ ^116^, ^117^ ^]^ Rather, the *V*_OC_ loss is determined by the relatively low radiative efficiency of PSCs, which stems from non‐radiative recombination both within the perovskite layers and at the perovskite/CTLs heterojunctions.

The influence of a CTL on *V*_OC_ is through its work function and the quality of the perovskite/CTL interface.^[^ ^91^ ^]^ A pure CTL is superior to its composite counterpart since the later perturbs the frontier energy levels and results in energy disorder.^[^ ^118^ ^]^ In addition, given the ionic defects at the perovskite/CTL interface, effective passivation (e.g., using quaternary ammonium halides^[^ ^89^ ^]^) is essential to reduce the charge trap density and prolong the carrier recombination lifetime for achieving low *V*_OC_ loss. Overall, the principles to achieve low *V*_OC_ loss can be summarized as follows: i) preparing high‐quality perovskite layers with high QFLS, ii) inserting proper interface layers to realize surface defect passivation, and iii) screening CTLs with excellent band alignment and reducing energy disorder in CTLs. We note that the most common strategy to reduce *V*_OC_ loss is to incorporate bulky cations into perovskites or modify the perovskite surface with them. In comparison, the former is inferior because incorporating bulky cations leads to the formation of the quasi 2D perovskite phase throughout the 3D perovskite layers, which may decrease the crystallinity of the 3D perovskite phase and reduces the device performance.^[^ ^119^ ^]^

## 3 Applications of WBG PSCs

To date, crystalline silicon solar cells (CSSCs) have accounted for over 90% of the photovoltaic market, and they have been applied in many fields for several decades. Thus, it is an arduous task for PSCs to supersede CSSCs, but PSCs still enjoy exceptional prospects in flexible devices, semi‐transparent devices, indoor photovoltaics, and tandem devices, and notably, the last three applications entail the use of high‐quality WBG perovskites.

### 3.1 Semitransparent Perovskite Solar Cells

Semitransparent PSCs (SPSCs), with both visible transparency and the ability to harness solar energy, have drawn unprecedented attention as they bear great promise for applications in building‐integrated photovoltaics (BIPV). In addition, SPSCs can absorb the albedo radiation from their surroundings, increasing the efficiency and lowering the system cost. To achieve efficient SPSCs, both the anode and cathode should be highly transparent to minimize the parasitic absorption, and the absorption layer should be semitransparent. We will not go into much detail about transparent electrodes because they have been deeply investigated and reviewed recently.^[^ ^120^, ^121^, ^122^ ^]^

Thus far, the feasible approaches to realize semi‐transparent perovskite layers are to deliberately make an ultrathin perovskite layer to achieve the required transparency or to fabricate microstructure within the perovskite films, such as by fabricating island‐ or mesh‐shaped perovskites;^[^ ^121^, ^123^ ^]^ however, these strategies bring two drawbacks: i) the thin perovskite films absorb less light and reduce shunt resistance, thus impairing the performance; ii) it is arduous to fabricate ultrathin or microstructured film during industrial production. By contrast, utilizing WBG perovskites as absorbers is a feasible strategy since they sufficiently absorb short‐wavelength light and let light beyond the absorption region pass through, which is important for achieving efficient SPSCs with high average transmittance (AVT).

Making the trade‐off between efficiency and transparency is the most critical subject for SPSCs. Yuan et al. combined modulation of Br^−^ content in WBG MAPbI_3−_ *_x_* Br*_x_* perovskites and thinner film to allow for high AVT and concomitantly achieved high PCE.^[^ ^124^ ^]^ The SPSCs with the optimal combination delivered a PCE of >10% with high transparency of 20%. If reduced perovskite film thickness is not utilized, only ≈2.3 eV perovskites are suitable to prepare SPSCs, because the photopic response of the human eye mainly ranges from 500 to 600 nm. In addition, the ≈2.3‐eV SPSCs are also compatible with BIPV for greenhouse applications. In this scenario, SPSCs harness the high‐energy, photosynthetically inactive photons while simultaneously letting the photosynthetically active photons pass through to maintain normal plant growth. Therefore, the pure bromide perovskites are preferred for the visual SPSCs. Our group utilized diphenyl ether as anti‐solvent to improve the crystallization process of MAPbBr_3_ perovskites.^[^ ^39^ ^]^ As a result, the trap‐state density of the MAPbBr_3_ film is reduced and the carrier lifetime is prolonged due to the enlarged crystal grains. Consequently, the PCEs of corresponding opaque and semitransparent PSCs were improved to 9.54% and 7.51%, respectively. The device devoid of a metal electrode displays 80% AVT in the wavelength range of 550–1000 nm, which, in theory, is sufficient for photosynthesis. Additionally, FAPbBr_3_ has exceptionally superior phase stability at elevated temperatures compared to its MA counterpart. Ying et al. fabricated FAPbBr_3_ films using a doctor‐blade‐coating process and found the addition of cetyltrimethyl ammonium bromide effectively inhibited the production of defects and achieved better uniformity.^[^ ^125^ ^]^ The FAPbBr_3_‐based opaque PSCs exhibited a PCE of 7.3%, and the corresponding SPSCs using silver nanowires as transparent electrodes achieved a PCE of 5.1% with AVT (400–800 nm) of 25%. Zuo et al. proposed ideal SPSCs that only absorb light in the UV and near‐infrared (NIR) regions while enabling most of the visible light to pass through, with the aim to attain high PCE and high AVT for human eyes.^[^ ^126^ ^]^ For the ultraviolet region, based on FAPbBr_3_, they developed FAPbBr_2.43_Cl_0.57_ perovskites with enlarged *E*_g_ (2.36 eV) and better morphology, which imparted a high V_OC_ of 1.55 V, a PCE of 7.5%, and an AVT up to 68% to the corresponding SPSCs. Then, they stacked the FAPbBr_2.43_Cl_0.57_ PSCs and NIR‐active organic photovoltaics to form a monoclinic tandem device, which exhibited a PCE of 10.7% with high AVT up to 52.9%.

### 3.2 Indoor‐Light Conversions

At present, requests for solar cells for the indoor photovoltaic market are becoming more numerous with the unprecedented development of the Internet of Things (IoT) because the portable IoT devices operating at the microwatt level usually need off‐grid self‐powered systems to ensure high portability.^[^ ^127^ ^]^ In particular, indoor photovoltaics enable the decrease of energy consumption inside a building by recycling photon energy from the interior lighting system. Indoor illumination generally ranges from 100 to 1000 lux, corresponding to an intensity of tens–hundreds µW cm^−2^, which is 2–3 orders of magnitude lower than STC and is more than sufficient for the electronic components in the IoT.

**Figure 4:**
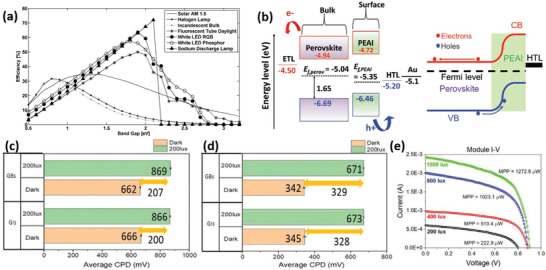
a) *E*_g_ versus the SQ limit under various source spectra; Reproduced with permission.^[^ ^137^ ^]^ Copyright 2021, Elsevier B. V. b) Energy band diagrams for the reference and PEAI‐deposited samples; average contact potential measured in the dark and under light for c) the reference and d) the PEAI‐deposited sample; e) *I–V* curves of the module using halogen light. Reproduced with permission.^[^ ^148^ ^]^ Copyright 2021, Wiley‐VCH.

Compared with solar cells operating outdoors, solar cells for indoor applications encounter three challenges. First, under such low‐intensity indoor light, the density of excess photogenerated carriers is significantly reduced, resulting in a relatively high ratio of trapped electrons to photo‐generated electrons. Then, even a low trap state density can cause significant energy loss by limiting quasi‐Fermi‐level splitting and shunting carriers, which is not a serious issue under STC;^[^ ^128^ ^]^ hence, the optimizations of indoor PSCs focus on modifying interfaces or judiciously choosing interface contacts to mitigate the interface trap state density.^[^ ^128^, ^129^, ^130^, ^131^, ^132^, ^133^, ^134^, ^135^, ^136^ ^]^ Second, indoor light sources exhibit distinct spectra limited to the visible wavelength range, and thus a device that is efficient under STC may not be the best option for harvesting indoor light due to the spectral discrepancy. The popular indoor light sources today are incandescent (halogen) lamps, fluorescent lamps, solid‐state LEDs, sodium discharge lamps, etc. **Figure** **4**a shows the theoretical PCE as a function of *E*_g_ calculated according to the SQ limit.^[^ ^137^, ^138^, ^139^ ^]^ The ideal *E*_g_ for the incandescent (halogen) lamp, fluorescent lamp/LED, and sodium discharge lamp are 0.8–0.9, 1.9–2.0, and 2.1 eV, respectively. The corresponding maximum PCEs are 30%, 50–64%, and 72%, respectively. Therefore, an optimal *E*_g_ of 1.9 eV was suggested for current indoor lighting sources, such as, fluorescent tubes or LEDs.^[^ ^140^ ^]^ Overall, *E*_g_ must be manipulated to harvest artificial light. Last, suitable interfacial layers and CTLs must be correspondingly tunable to match with the perovskites and improve the carrier extraction.

Obviously, PSCs are particularly interesting for indoor applications due to their following primary advantages: i) the altered *E*_g_ is in favor of achieving an optimum efficiency by minimizing energy loss of relaxation; ii) compatibility of PSC fabrication with printing technologies allows extremely low‐cost manufacture and integration of industrially printed electronic devices.^[^ ^141^ ^]^ iii) the benign indoor environment can largely extend the lifetimes of PSCs.^[^ ^134^ ^]^ As a significant example, heavily Br‐doped perovskites are photo‐stable under indoor illumination since the dim light cannot drive phase segregation.^[^ ^142^ ^]^

MAPbI_3_ PSCs were evaluated early for use with the indoor light illumination, and a promising high PCE up to 27.4% was obtained under fluorescent lamp illumination at 100–1000 lux benefiting from the passivation effect of PCBM.^[^ ^143^ ^]^ This result makes PSCs potentially valuable for harvesting indoor light and supporting IoT elements. Further optimizations of PSCs for indoor applications are predominantly concentrated on reinforcing the rectification effect and increasing shunt resistance.^[^ ^129^, ^130^, ^131^, ^132^ ^]^ However, as mentioned, most of the indoor lamps exhibit illumination wavelengths from 350 to 750 nm, and MAPbI_3_ will lead to thermal loss in PSCs due to the unsuitable *E*_g_. Lim et al. prepared MAPbBr*_x_* I_3−_ *_x_* perovskites for LED illumination and found that 10% Br doping confers perovskites (*E*_g_ = 1.63 eV) with larger grains and reduced trap sites, whereas a Br/I ratio over 10% for further widening *E*_g_ reduces the grain sizes and brings about more trap states.^[^ ^144^ ^]^ Consequently, the 10% Br‐doped PSCs examined under LED illumination (1000 lux) exhibited an average PCE of 34.5% ±1.2%. To break the barrier of 10% Br doping, Cs^+^ or Cl^−^ in MAPbBr*_x_* I_3−_ *_x_* perovskite systems can be used to achieve wider bandgap while ensuring high‐quality films by controlling the crystallization process.^[^ ^145^, ^146^ ^]^ Cheng et al. prepared MAPbI_2−_ *_x_* BrCl*_x_* (*x* ≈ 30%, *E*_g_ = 1.8 eV) for PSCs and achieved a high PCE of 36.2% with large *V*_OC_ of 1.028 V under 1000 lux fluorescent light, although the device reached a PCE of only 14.6% under STC.^[^ ^146^ ^]^ Note that a high PCE of PSCs under STC does not assure their excellent performance under indoor light conditions.

To further deepen the ionic mixing engineering research, two similar‐composition perovskites of (FA_0.6_MA_0.4_)_0.9_Cs_0.1_Pb(I_0.6_Br_0.4_)_3_ and Cs_0.05_(FA_0.6_MA_0.4_)_0.95_Pb(I_0.6_Br_0.4_)_3_ (*E*_g_ = 1.75 eV) were proposed successively.^[^ ^147^, ^148^ ^]^ For this composition, increasing the Br ratio to 0.5 will extend *E*_g_ to 1.88 eV, meeting the optimal *E*_g_ of indoor light applications. In contrast, excessive Br deteriorated the film quality, leading to pinholes that degrade the device performance. Therefore, the Br ratio was reduced to 0.4 to achieve a balance between *E*_g_ and film quality. In the above studies, PEAX (X = I, Br, and Cl) was used to passivate the perovskite surface, thus suppressing the non‐radiative recombination and reducing the *V*_OC_ loss. As a result, PSCs based on the (FA_0.6_MA_0.4_)_0.9_Cs_0_ *_._*_1_Pb(I_0_ *_⋅_*_6_Br_0.4_)_3_^[^ ^147^ ^]^ and Cs_0.05_(FA_0.6_MA_0.4_)_0.95_Pb(I_0.6_Br_0.4_)_3_^[^ ^148^ ^]^ reached indoor PCEs of 35.6% (white LED, 1000 lux) and 33.4% (white LED, 200 lux), respectively. In particular, PEAI modification fosters the formation of a thin hole‐selective perovskite layer on the surface of the original film that results in higher charge collection (Figure 4b) and eliminates the contact potential difference discrepancy between the grain interiors and the grain boundaries, thus forming homogeneous charge separation (Figure 4c,d). Strikingly, an indoor module containing 9 devices of 25 mm^2^ area connected in parallel was fabricated, and, as per Figure 4e, it produced maximum powers of 223, 510, 1023, and 1273 µW at 200, 400, 800, and 1000 lux, respectively, under a halogen light. These power outputs are sufficient to run low‐power wireless protocols. The characterization of PSCs under dim indoor light is susceptible to operating conditions, such as non‐parallelism of indoor light, stray light, and uncalibrated indoor light intensity.^[^ ^149^ ^]^ Noticeably, the pronounced hysteresis—more severe than that in the case of STC—may lead to grossly overestimated PCE measured under indoor light because the capacitance current in the hysteresis effect is comparable to the photocurrent at low light intensity.^[^ ^132^, ^136^, ^142^, ^150^ ^]^

### 3.3 Tandem Solar Cells

Multijunction tandem solar cells (TSCs) utilizing a combination of at least two solar cells stacked vertically maximally absorb the incident sunlight and minimize the thermal losses, surpassing the 33.7% from the single‐junction counterpart.^[^ ^115^, ^151^ ^]^ Taking the binary junction as an example, TSCs can be categorized into four‐terminal (4T) devices and two‐terminal (2T) devices. 4T devices consist of two mechanically stacked independent solar cells, while the monolithic 2T device is constructed in series on a single substrate. The 2T device requires more sophisticated optimization to achieve current matching by altering absorption layer thicknesses and *E*_g_s. As an example, a 1.2 eV rear cell requires a top cell of 1.75–1.85 eV for the 2T architecture, whereas the 4T architecture has a relaxed requirement of 1.6–1.9 eV. Notably, in theory, the 2T and 4T architectures may show almost identical efficiency values (<1.5% absolute difference).^[^ ^152^ ^]^ Thus far, efficient TSCs have been assembled by combining WBG perovskite top cells with various rear cells involving CSSCs, inorganic thin‐film solar cells utilizing CIGS solar cells, or GaAs, NBG PSCs, and organic solar cells (OSCs).

#### 3.3.1 Perovskite/Silicon TSCs

Further promoting the current maximum PCE of 25.5% is arduous since this value is already extremely close to the theoretical maximum efficiency of 29.4%.^[^ ^153^, ^154^ ^]^ Nevertheless, raising PCE is the key driver for reducing the levelized cost of electricity generated by photovoltaics after taking the balance‐of‐systems cost into consideration. Since CSSC is a promising bottom cell candidate as it provides excellent NIR spectral response, designing perovskite/silicon TSCs is regarded as an effective approach to increase energy yield. Meanwhile, this concept enables PSCs to effortlessly capitalize upon the existing photovoltaics industry chain.^[^ ^155^ ^]^

**Figure 5:**
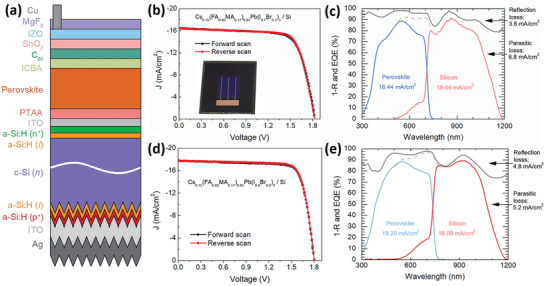
a) Schematic structure of perovskite/Si TSCs. *J–V* curves of b) Cs_0.15_(FA_0.83_MA_0.17_)_0.85_Pb(I_0.7_Br_0.3_)_3_/Si and d) Cs_0.15_(FA_0.83_MA_0.17_)_0.85_Pb(I_0.8_Br_0.2_)_3_/Si TSCs. EQE and total absorbance of the c) Cs_0.15_(FA_0.83_MA_0.17_)_0.85_Pb(I_0.7_Br_0.3_)_3_/Si and e) Cs_0.15_(FA_0.83_MA_0.17_)_0.85_Pb(I_0.8_Br_0.2_)_3_/Si TSCs. Reproduced with permission.^[^ ^159^ ^]^ Copyright 2019, Elsevier B. V.

Considering the ideal current match, a 1.70–1.85‐eV top cell is the best partner for ≈1.1‐eV CSSCs.^[^ ^156^ ^]^ However, the realistic parasitic absorption and reflection caused by other functional layers will drop *J*_SC_ of the top PSC. Hence, especially in 2T TSCs, perovskites with 1.6–1.7 eV bandgap are preferred to compensate for the insufficient current at present.^[^ ^157^, ^158^ ^]^ A representative result has been given by Chen et al.^[^ ^159^ ^]^ They used a combination of MACl and MAH_2_PO_2_ additives to enlarge the grain size and passivate the grain boundaries of WBG perovskite films. The PSCs based on Cs_0.15_(FA_0.83_MA_0.17_)_0.85_Pb(I_0.7_Br_0.3_)_3_ (*E*_g_ = 1.70 eV) yielded a PCE of 18.6%, with a high *V*_OC_ of 1.19 V. The corresponding 2T TSCs (**Figure** **5**a) delivered a PCE of 22.4% coupled with a high *V*_OC_ of 1.83 V and a small *J*_SC_ of 16.4 mA cm^−2^ (Figure 5b,c). The low current density was caused by insufficient absorption, parasitic absorption in the electrodes and CTLs, and a strange blue shift of the EQE edge. To compensate for the *J*_SC_ loss, they had to reduce *E*_g_ to 1.64 eV, and then the corresponding TSCs attained a high PCE of 25.4% with a *V*_OC_ of 1.80 V and a *J*_SC_ > 18 mA cm^−2 ^(Figure 5d,e). Overall, *E*_g_ > 1.7 eV is not conducive to high‐performance in 2T TSCs at present,^[^ ^160^ ^]^ whereas the scenario is improved in 4T TSCs because they are insensitive to current matching.

As mentioned, in 2016, McMeekin et al. proposed FA_0.83_Cs_0.17_Pb(I_0.6_Br_0.4_)_3_ (*E*_g_ = 1.74 eV) perovskites with enhanced photostability and, PSCs based on it yielded a PCE of 17.1%.^[^ ^74^ ^]^ By combining it with a 19% CSSC, 4T TSCs achieved a PCE > 25%. Motivated by this work, relevant research was undertaken focused on pursuing high‐quality WBG perovskite films for tandem applications. Duong et al. demonstrated Rb^+^ doping could improve the crystallinity, suppress defect migration, and improve light stability for FA_0.75_MA_0.15_Cs_0.1_PbI_2_Br (*E*_g_ = 1.73 eV) perovskites. The corresponding opaque PSCs and SPSCs showed stabilized PCEs of 17.4% and 16.0%, respectively, as well as negligible hysteresis and excellent AVT of ≈84% between 720 and 1100 nm. The corresponding 4T TSCs incorporating a 23.9% CSSC yielded a PCE of 26.4%.^[^ ^161^ ^]^ Then they further passivated the perovskite defects by forming a quasi‐2D BABr‐based perovskite phase on the surface. The champion device showed an improved efficiency of 18.3% with a substantial increased *V*_OC_ to 1.26 V. Based on this, the PCE of 4T TSCs was increased to 27.7%.^[^ ^119^ ^]^

#### 3.3.2 Perovskite/CIGS and Perovskite/GaAs TSCs

**Figure 6:**
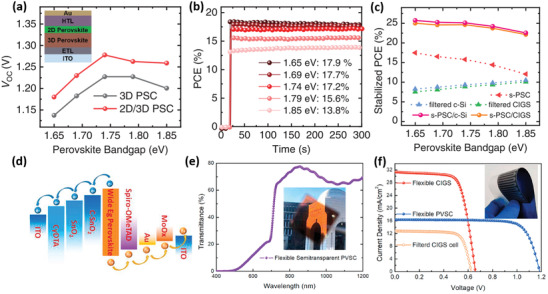
a) *V*_OC_ and b) stabilized PCE versus the perovskite *E*_g_ for opaque PSCs; c) stabilized PCE of a SPSC, filtered CSSC, filtered CIGS solar cell, and the corresponding 4T TSCs (calculated). Reproduced with permission.^[^ ^164^ ^]^ Copyright 2020, Wiley‐VCH. d) Energy level alignment diagram of the SPSC; e) transmittance spectrum of the SPSC; f) *J–V* curves of 4T, all‐flexible, perovskite/CIGS TSCs. Reproduced with permission.^[^ ^165^ ^]^ Copyright 2020, Elsevier B. V.

CIGS solar cells are also able to pair with PSCs in TSCs. The short‐wavelength response (<600 nm) of CIGS is insufficient, mainly due to the optical loss from the CdS layers. Therefore, the EQE derived from PSCs and filtered CIGS exceeds that of the independent CIGS device. Given the widely tunable *E*_g_ from 1.0 to 1.7 eV of CIGS, the ideal rear and front sub‐cells to achieve maximum PCE should have *E*_g_ of 1.1 eV for CIGS and 1.7 eV for perovskite, the combination of which is predicted to attain a high PCE of over 30% according to optical simulation.^[^ ^162^ ^]^ Earlier, Teodor et al. ventured to prepare perovskite/CIGS 2T TSCs where the *E*_g_ of CIGS was set as 1.04 eV and a series of MAPbI_3−_ *_x_* Br*_x_* absorbers with *E*_g_ from 1.58 to 2.29 eV was obtained via vapor‐based halide exchange reactions. This work demonstrated the feasibility of perovskite/CIGS 2T TSCs, although the TSCs only attained a PCE of 10.9% while using a 1.72 eV perovskite layer.^[^ ^163^ ^]^ Then, based on the shared Cs_0.17_FA_0.83_PbI_1.8_Br_1.2_ perovskites, Shen et al. developed quarter‐cation Cs/Rb/MA/FA WBG perovskites that lead to negligible hysteresis and much‐reduced sensitivity toward oxygen exposure for PSCs.^[^ ^162^ ^]^ The SPSCs based on Cs_0.05_Rb_0.05_FA_0.765_MA_0.135_PbI_2.55_Br_0.45_ (*E*_g_ = 1.62 eV) and Cs_0.10_Rb_0.05_FA_0.75_MA_0.15_PbI_1.8_Br_1.2_ (*E*_g_ = 1.75 eV) attained PCEs of 18.1% and 16.0%, respectively. They enable PCEs of 23.9% and 23.4% for 4T TSCs formed by combination with a 16.5% CIGS cell. In further, Gharibzadeh employed BABr to modify the WBG FA_0.83_Cs_0.17_PbI_3−_ *_y_* Br*_y_* perovskite surface, and the formed 2D/3D heterostructure reduced the non‐radiative recombination and gave a strong enhancement in *V*_OC_ of PSCs (**Figure** **6**a).^[^ ^164^ ^]^ As per Figure 6b, all PSCs delivered high stabilized PCEs of 17.9%, 17.6%, 17.2%, 15.6%, and 13.8% with increasing *E*_g_ from 1.65 to 1.69, 1.74, 1.79, and 1.85 eV. Then based on the 1.65 eV SPSCs, stabilized PCEs of up to 25.7% and 25.0% in 4T perovskite/c‐Si and perovskite/CIGS TSCs were obtained, respectively. It is worth noting that, as per Figure 6c, similar stabilized PCEs are observed when the 1.69 and 1.74 eV PSCs were adopted, while further increasing *E*_g_ will decrease the PCE of TSCs. In addition, highly efficient perovskite/CIGS TSCs were reported by using the *E*_g_ < 1.7 eV PSCs, and as an example, a PCE of 25.9% has been obtained in 4T TSCs using 1.68 eV PSCs.^[^ ^166^ ^]^ Thus, there is still much room for improving the PCE of perovskite/CIGS TSCs.

Another advantage of perovskite/CIGS TSCs is their compatibility with flexible devices. Li et al. fabricated efficient, flexible, FA_0.8_Cs_0⋅2_Pb(I_0⋅7_Br_0.3_)_3_ SPSCs by constructing gradient energy‐level alignments, as per Figure 6d, where a triple‐layer ETL with a gradient Fermi‐level alignment and an HTL/Au/MoO_3_ structure was designed to facilitate effective charge transport.^[^ ^165^ ^]^ The champion flexible 1.75‐eV SPSC achieved a PCE of 15.0% with superb light transmittance of ≈70% beyond 700 nm wavelength (Figure 6e) and, as per Figure 6f, allowing all‐flexible perovskite/CIGS 4T TSCs with PCE beyond 21%.

GaAs solar cells possess various advantages such as a low temperature coefficient, excellent low‐light performance, and high PCE approaching the SQ limit. GaAs (*E*_g_ = 1.4 eV) has been successfully utilized in 2T TSCs by combining it with InGaP (*E*_g_ = 1.80–1.90 eV) solar cells. However, this combination will boost the overall manufacturing cost and a complicated III–V tunnel‐junction diode to connect the subcells is inevitable. Therefore, substituting InGaP with perovskites is of significance for GaAs solar cells. Unlike other solar cells, GaAs solar cells require perovskites with *E*_g_ > 1.8 eV for tandem fabrication, and such wide *E*_g_ requires a Br content of ≈50%, which will lead to pinholes in the film as mentioned previously, even in the FA_0.80_Cs_0.20_Pb(I_0.50_Br_0.50_)_3_ system. In this regard, Li et al. used a solvent‐evaporation‐annealing process to improve the morphology and MA incorporation to increase the defect tolerance and photostability against halide segregation.^[^ ^167^ ^]^ The SPSCs based on FA_0.80_MA_0.04_Cs_0.16_Pb(I_0.50_Br_0.50_)_3_ (*E*_g_ = 1.82–1.89 eV) perovskites delivered a PCE of 14.1% while illuminated from the transparent electrode side. With these SPSCs as top cells, the PCE of GaAs solar cells was further improved from 21.7% (bulk GaAs solar cell) to 24.3% (2T) and 25.2% (4T). Moreover, this approach is also suitable for thin‐film GaAs solar cells. The obtained flexible TSCs yielded a PCE of 24.3% and superior bending resistance (98% of initial efficiency after 1000 bending cycles).

#### 3.3.3 All‐Perovskite TSCs

Concerning perovskite/Si, CIGS,or GaAs TSCs, energy‐intensive processes are involved in the fabrication, despite the promising results. In comparison, all‐perovskite TSCs deserve more effort due to the simpler fabrication processes and potentially low material cost. The theoretical maximum efficiency of 46.1% is estimated for all‐perovskite TSCs comprising perovskite materials of 0.9–1.2 eV and 1.7–1.9 eV for the bottom and top cells, respectively, surpassing evidently the 33.7% from a single‐junction counterpart.^[^ ^74^, ^168^ ^]^ The early example of all‐perovskite TSCs was fabricated by sandwiching subcells based on MAPbBr_3_ and MAPbI_3_, which were laminated with a thick, doped HTL. Despite the *V*_OC_ as high as 2.2 V, the PCE was limited to 10.4% due to poor light management.^[^ ^169^ ^]^ Sheng et al. estimated the feasible efficiency of a device utilizing this *E*_g_ combination to be 21.3%. The estimated PCE can further increase to 25.9% by integrating MAPbI_3_ with a 2.05‐eV perovskite.^[^ ^170^ ^]^ Forgács et al. presented efficient 2T TSCs by pairing a Cs_0.15_FA_0.85_Pb(I_0.3_Br_0.7_)_3_ (*E*_g_ ≈ 2 eV) perovskite with MAPbI_3_, which delivered a record PCE of 18%.^[^ ^171^ ^]^ Apparently, the mismatch of the perovskite absorbers is one factor limiting the overall PCE.

The flourishing development of high‐quality Sn/Pb alloyed perovskite materials of *E*_g_ ≈ 1.2 eV benefits the building of all‐perovskite TSCs, and pursuing perovskites with complementary *E*_g_ becomes more critical to maximize the light‐harvesting region of TSCs. Eperon et al. developed a 1.2‐eV FA_0.75_Cs_0.25_Sn_0.5_Pb_0.5_I_3_ perovskite, and PSCs based on it can deliver 14.8% efficiency. Combining it with FA_0.83_Cs_0.17_Pb(I_0.5_Br_0.5_)_3_ (*E*_g_ = 1.8 eV) resulted in 2T TSCs yielding PCE of 17.0% with 1.65 V *V*_OC_.^[^ ^172^ ^]^ In the following research, the combination of Pb/Sn NBG perovskites and FA/Cs/Pb/I/Br WBG perovskites dominated the TSCs. **Table** **1** summarizes the PCE of all‐perovskite TSCs employing WBG > 1.7 eV perovskites. Thanks to the synergetic development of NBG perovskites and excellent recombination layers,^[^ ^173^, ^174^, ^175^, ^176^, ^177^, ^178^, ^179^ ^]^ the PCE of all‐perovskite TSCs has exceeded 24%.

**Table 1:** The PCE of all‐perovskite TSCs employing WBG perovskites >1.7 eV

Type	WBG perovskite	NBG perovskite	*E*_g_s	PCE [%]	Ref
2T	MAPbBr_3_	MAPbI_3_	2.25/1.55 eV	10.4	^[^ ^169^ ^]^
2T	Cs_0.15_FA_0.85_Pb(I_0.3_Br_0.7_)_3_	MAPbI_3_	2/1.55 eV	18.1	^[^ ^171^ ^]^
2T	FA_0.83_Cs_0.17_Pb(I_0.5_Br_0.5_)_3_	FA_0.75_Cs_0.25_Sn_0.5_Pb_0.5_I_3_	1.8/1.2 eV	17.0	^[^ ^172^ ^]^
2T	MA_0.9_Cs_0.1_Pb(I_0.6_Br_0.4_)_3_	MAPb_0.5_Sn_0.5_I_3_	1.8/1.2 eV	18.5	^[^ ^173^ ^]^
2T	Cs_0.05_FA_0.8_MA_0.15_PbI_2.55_Br_0.45_	(FASnI_3_)_0.6_(MAPbI_3_)_0.4_	1.75/1.25 eV	23.4	^[^ ^174^ ^]^
2T	FA_0.8_Cs_0.2_Pb(I_0.7_Br_0.3_)_3_	(FASnI_3_)_0.6_(MAPbI_3_)_0.4_	1.75/1.25 eV	21.0	^[^ ^175^ ^]^
2T	FA_0.6_Cs_0.4_Pb(Br_0.3_I_0.7_)_3_	FA_0.75_Cs_0.25_Sn_0.5_Pb_0.5_I_3_	1.76/1.26 eV	19.1	^[^ ^176^ ^]^
2T	FA_0.6_Cs_0.4_Pb(I_0.65_Br_0.35_)_3_	FA_0.5_MA_0.45_Cs_0.05_Pb_0.5_Sn_0.5_I_3_	1.80/1.22 eV	22.7	^[^ ^177^ ^]^
2T	FA_0.6_Cs_0.3_DMA_0.1_PbI_2.4_Br_0.6_	FA_0.75_Cs_0.25_Sn_0.5_Pb_0.5_I_3_	1.7/1.26 eV	23.1	^[^ ^96^ ^]^
2T	FA_0.83_Cs_0.17_Pb(Br_0.7_I_0.3_)_3_	MAPbI_3_	1.94/1.57 eV	15.0	^[^ ^180^ ^]^
2T	Cs_0.4_FA_0.6_PbI_1.95_Br_1.05_	Cs_0.05_MA_0.45_FA_0.5_Pb_0.5_Sn_0.5_I_3_	1.78/1.21 eV	24.4	^[^ ^178^ ^]^
4T	FA_0.8_Cs_0.2_Pb(I_0.7_Br_0.3_)_3_	(FASnI_3_)_0.6_(MAPbI_3_)_0.4_	1.75/1.25 eV	23.0	^[^ ^179^ ^]^
2T	FA_0.8_Cs_0.2_Pb(I_0.6_Br_0.4_)_3_	FA_0.7_MA_0.3_Pb_0.5_Sn_0.5_I_3_	1.77/1.22 eV	24.8	^[^ ^181^ ^]^

**Figure 7:**
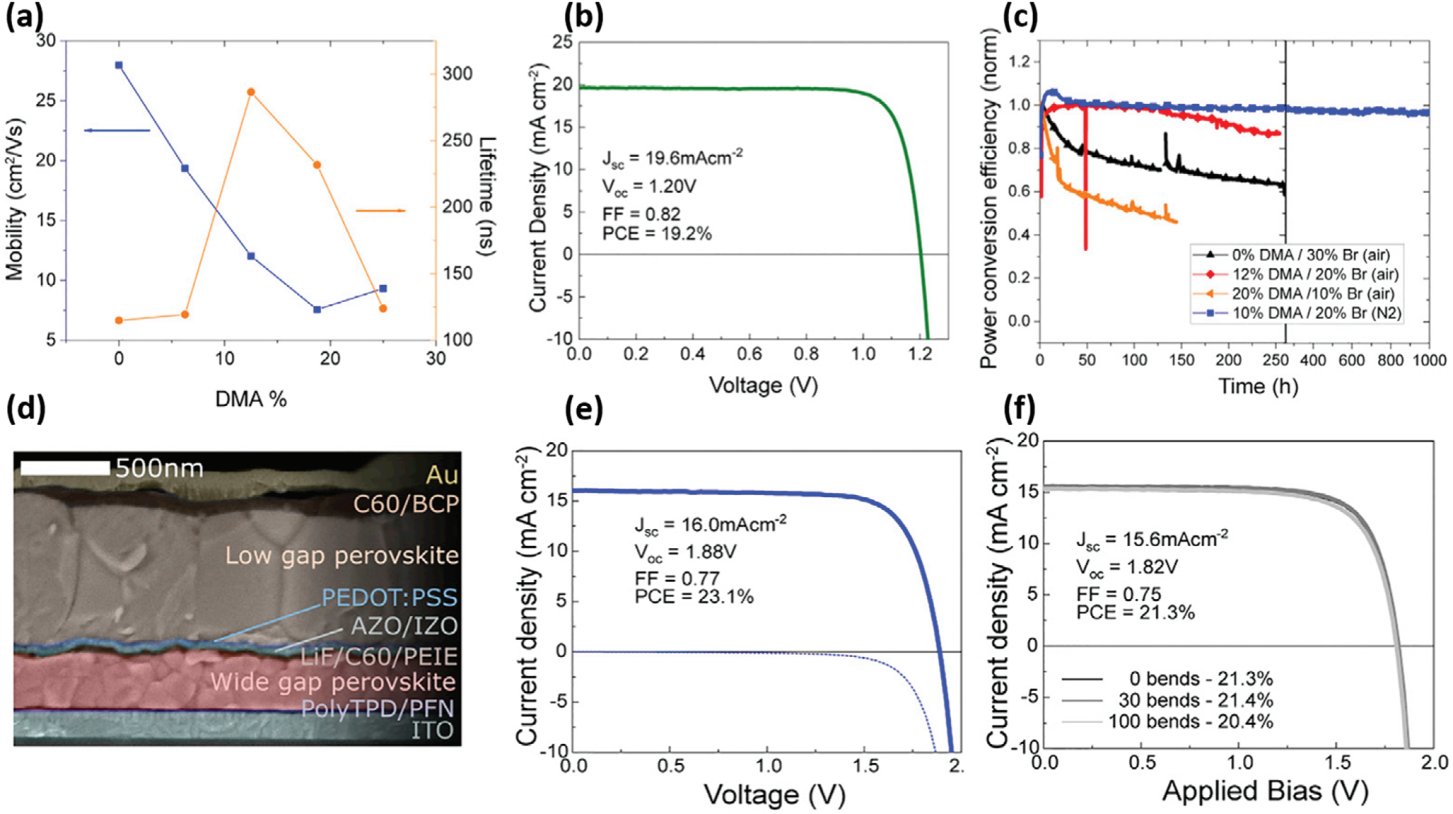
a) Mobility and carrier lifetime versus DMA percentage; b) *J–V* curves of the champion DMA‐containing device; c) long‐term stability of PSCs with various DMA and Br percentages; d) cross‐sectional SEM of an all‐perovskite TSC. *J–V* curves for e) rigid and f) flexible all‐perovskite 2T tandem devices. Reproduced with permission.^[^ ^96^ ^]^ Copyright 2019, Elsevier B. V.

Specially, Palmstrom et al. mixed bulky dimethylammonium (DMA) cations in WBG perovskites to reduce the Br usage.^[^ ^96^ ^]^ As per **Figure** **7**a, although the addition of DMA decreases the charge‐carrier mobility, the formed FA_0.6_Cs_0.3_DMA_0.1_PbI_2.4_Br_0.6_ (*E*_g_ = 1.7 eV) displayed a prolonged lifetime and suppressed halide segregation due to the reduction of defect density. The PSCs based on FA_0.6_Cs_0.3_DMA_0.1_PbI_2.4_Br_0.6_ attained a champion PCE of ≈19% and exhibited excellent operational stability (Figure 7b,c).

Then this device was combined with a NBG FA_0.75_Cs_0.25_Sn_0.5_Pb_0.5_I_3_ device with a PCE of 16.5% to obtain an all‐perovskite rigid 2T TSC (Figure 7d), which exhibited a stable PCE of up to 23.1% with a *V*_OC_ of 1.88 V (Figure 7e). Further, taking advantage of the low‐temperature tandem processing, as per Figure 7f, the fabricated all‐perovskite flexible TSC delivered a steady PCE of 21.3%, which came down to 20.4% after 100 bends at 1.5 cm bend radius.^[^ ^96^ ^]^

In addition to the double‐junction TSCs, triple‐junction TSCs were also proposed benefiting from the tunable *E*_g_ and excellent interconnection layers. McMeekin et al. first realized all‐solution‐processed triple‐junction TSCs by utilizing the extremely volatile acetonitrile (CH_3_CN)/MA mixed solvent to prepare perovskites.^[^ ^180^ ^]^ The 2T TSCs utilizing FA_0.83_Cs_0.17_Pb(Br_0.7_I_0.3_)_3_ (*E*_g_ = 1.94 eV), MAPbI_3_ (*E*_g_ = 1.57 eV), and MAPb_0.75_Sn_0.25_I_3_ (*E*_g_ = 1.34 eV) reached a *V*_OC_ of 2.83 V, yet a low PCE of 6.7%. Through optical and electronic simulation, the PCE of a triple‐junction device was estimated to be 26.7%. Wang et al. reported a triple‐junction TSCs with a PCE above 16.8% by combining high‐quality Cs_0.1_(FA_0.66_MA_0.34_)_0.9_PbI_2_Br (*E*_g_ = 1.73 eV), FA_0.66_MA_0.34_Pb‐ I_2.85_Br_0.15_ (*E*_g_ = 1.57 eV), and FA_0.66_MA_0.34_Pb_0.5_Sn_0.5_I_3_ (*E*_g_ = 1.23 eV);^[^ ^182^ ^]^ however, this triple‐junction TSC was inferior to the double‐junction device employing the combination of Cs_0.1_(FA_0.66_MA_0.34_)_0.9_PbI_2_Br and FA_0.66_MA_0.34_Pb_0.5_Sn_0.5_I_3_, as the latter yielded a PCE of 19.8%. In addition to reducing *J*_SC_ loss originating from parasitic absorption and incomplete light absorption of NBG perovskite, further improving the PCE of triple‐junction TSCs urgently requires an efficient ≈2‐eV *E*_g_ perovskite with low *V*_OC_ loss.

#### 3.3.4 Perovskite/Organic TSCs

Perovskite/organic TSCs are more appealing than all‐perovskite TSCs, even though they are both compatible with simple solution processing and flexible substrates, because the former not only enjoys the orthogonal solvent systems that can reduce the challenge of constructing interconnecting layers but also possesses no oxidation stability issue that occurs in NBG perovskite films. The OSCs usually employ absorbers with *E*_g_ of 1.2–1.3 eV, matching well with CsPbI_2_Br perovskite (*E*_g_ = 1.92 eV).^[^ ^183^, ^184^, ^185^, ^186^, ^187^ ^]^ However, inorganic perovskites require a high‐temperature annealing process, which is incompatible with flexible substrates and energy conservation.

**Figure 8:**
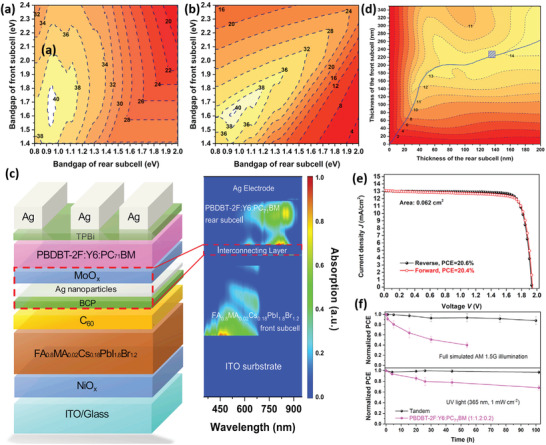
PCE limits of a) 4T and b) 2T TSCs; c) structure and the photon absorption distributions of TSCs; d) *J*_SC_ versus the thicknesses of front and rear subcells; e) *J–V* curves of the champion TSC. f) Photostability and UV sensitivity tests of TSCs and OSCs. Reproduced with permission.^[^ ^191^ ^]^ Copyright 2020, Elsevier B. V.

Turning to organic–inorganic hybrid perovskites, combining the preliminary explorations of perovskite/organic TSCs,^[^ ^188^, ^189^, ^190^ ^]^ Chen et al. established a semi‐empirical model for perovskite/organic TSCs to assess the PCE limit of various combinations.^[^ ^191^ ^]^ As shown in **Figure** **8**a,b, the PCE of both the 4T and 2T TSCs can exceed 40%. Accordingly, a Y6‐based OSC (*E*_g_ = 1.41 eV) was selected as the rear cell since it generally exhibited average EQE of 80%, high FF, and small *V*_OC_ loss. Although a 1.95‐eV PSC is theoretically more suitable for Y6‐based rear cells, considering the actual performance level of WBG PSC, the most suitable *E*_g_ is actually ≈1.75 eV. They fabricated 2T perovskite/organic TSCs employing FA_0.8_MA_0.02_Cs_0.18_PbI_1.8_Br_1.2_ (≈230 nm, *E*_g_ = 1.77 eV) perovskite for front subcells and NBG OSC (≈135 nm, *E*_g_ = 1.41 eV) for rear subcells, forming the configuration ITO/NiO*_x_*/perovskite/C_60_/BCP/Ag/MoO*_x_*/PBDBT‐2F:Y6:PCBM/TPBi/Ag shown in Figure 8c. Upon optimizing the NiO*_x_* HTL structure, interconnecting layer, and active layer thickness (Figure 8d), the best TSC showed a remarkable PCE of 20.6% with a *V*_OC_ of 1.902 V and a *J*_SC_ of 13.05 mA cm^−2^ (Figure 8e). In addition, the perovskite front cell also acts as the UV filter for the organic rear cell, thus improving the photostability (Figure 8f). Li et al. developed efficient perovskite/organic 2T TSCs integrating Cs_0.1_(FA_0.6_MA_0.4_)_0.9_Pb(I_0.6_Br_0.4_)_3_ perovskite (*E*_g_ = 1.74 eV) top subcells and PBDB‐T:SN6IC‐4F (1.30 eV) bottom subcells.^[^ ^90^ ^]^ Upon passivating the perovskite surface with PMABr, the resulting rigid and flexible perovskite/organic TSCs showed remarkable PCEs of 15.1% and 13.6%, with corresponding *V*_OC_ of 1.85 and 1.80 V, respectively. Moreover, a photovoltaic‐driven electrolysis system combining the TSCs and water splitting electrocatalysis was assembled, demonstrating a solar‐to‐hydrogen efficiency of 12.3% and 11.2% for rigid, and flexible perovskite/organic TSCs. Strikingly, Brinkmann et al. declared that they selected FA_0.8_Cs_0.2_Pb(I_0.5_Br_0.5_)_3_ (*E*_g_ = 1.85 eV) perovskite and a [2‐(3,6‐dimethoxy‐9H‐carbazol‐9‐yl)ethyl]phosphonic acid (MeO‐2PACz) HTL to prepare PSCs, which provided a high stabilized *V*_OC_ of 1.34 V and a high PCE of 16.8% upon combining with PEAI passivation.^[^ ^192^ ^]^ Then the corresponding TSCs assembled by further stacking PSCs with PM6:Y6:PCBM OSCs and optimizing the interconnecting layers yielded a PCE of 23.5% accompanied by a *V*_OC_ of 2.15 V. This result enabled perovskite/organic TSCs to be on par with perovskite/CIGS and all‐perovskite multi‐junctions.

#### 3.3.5 Evaluations of Various TSCs

Thus far, the champion PCEs of various TSCs are still far below the theoretical estimations (>30%)^[^ ^193^ ^]^ and are restrained by several dominating factors: i) untight integration and undesirable electrical management of the connecting recombination layers, ii) large parasitic absorption loss of other functional layers, iii) large current loss in the NBG subcells, and iv) large *V*_OC_ loss in the WBG subcells. In detail, perovskite/Si and perovskite/CIGS TSCs currently require optimal perovskites of *E*_g_ ranging from 1.60 to 1.70 eV, caused by the combination of (ii) and (iv). Therefore, potentially higher PCEs of perovskite/Si and perovskite/CIGS TSCs can be achieved by combining them with >1.7‐eV PSCs after reducing the parasitic absorption and *V*_OC_ loss. As for the all‐perovskite TSCs, although (iv) is still the main problem restricting the performance, it is limited more by the NBG perovskites suffering from stability issues due to the easy oxidization of Sn^2+^. Meanwhile, it is challenging to obtain a thick Pb‐Sn mixed perovskite film while maintaining excellent photoelectric properties; thus, the current density deficit in NBG PSCs is also non‐negligible. In particular, all‐perovskite TSCs entail compact recombination layers that prevent the re‐dissolution of the layers below. Although orthogonal solvent and solvent‐free (e.g., thermal evaporation) approaches can be adopted to prepare the second perovskite layer, to date, only simple perovskite compositions have been prepared using these methods. In contrast, more complex perovskite compositions are required to realize WBG and desired PCE. The perovskite/organic TSCs enjoy lower requirements of the recombination layers due to the orthogonal solvents, while they are limited by (iii), that is, the low PCE of OSCs as well as insufficiently long absorption wavelength. Importantly, (iv), large *V*_OC_ loss in the WBG subcells, is a severe, yet shared, constraint for all the TSCs.

In terms of the development perspective, perovskite/CIGS or GaAs stacking is inferior to the other combinations as the high manufacturing costs hinder their large‐scale deployment. In contrast, the perovskite/Si pair is the most easily acceptable due to the complete product lines of CSSCs. Additionally, both the all‐perovskite TSCs and perovskite/organic TSCs are brand‐new tandem systems, and they fully retain the advantages of the perovskite system, including low cost, high throughput, high specific power, and compatibility with flexible substrates in particular.

## 4 Conclusion and Outlook

In summary, we have systematically reviewed the extensive developmental progress according to the various compositions of WBG perovskite materials in this paper. Researchers have developed a variety of optimization strategies for WBG perovskite materials, including mixed‐cation engineering, additive engineering, interface‐modification engineering, etc. and revealed the influencing mechanisms of various optimizations. Therefore, the WBG PSCs have developed rapidly, with PCE close to 20%, and the criticized photo‐instability issue has been significantly relieved. In addition, we systematically discussed the applications of WBG PSCs in the fields of semi‐transparent devices, low‐light indoor applications, and TSCs. WBG PSCs are very promising either as stand‐alone devices or in a tandem structure with NIR active devices.^[^ ^194^ ^]^

Despite the significant progress described above, the investigation of WBG perovskites is still in its infancy, and the PCE loss is still relatively larger compared with TBG perovskites. A series of scientific issues involving the crystallization mechanism, phase segregation, ion migration, *V*_OC_ loss, and the fatigue effect should be resolved by combining experimental and theoretical methodologies. Such understanding will lay the foundation for deliberately optimizing WBG perovskites. Undoubtedly, WBG PSCs have emerged as one of the powerful branches to extend the technical directions of perovskites. In view of the market position of CSSCs and the unique application fields of WBG PSCs, it is believed that WBG perovskites will play a dominant (or more dominant than TBG perovskites) role in shaping the development of perovskite technologies and accelerating the industrialization process.

## Conflict of Interest

The authors declare no conflict of interest.

## Supporting information

Supporting Information
