# Supplementary material for: Wide‐Bandgap Organic–Inorganic Lead Halide Perovskite Solar Cells
Source: Adv Sci (Weinh). 2022 Mar 8;9(14):2105085. doi: 10.1002/advs.202105085 (PMC9109050; doi:10.1002/advs.202105085)
Supplement: Supplementary file 1 — Supporting Information [file ADVS-9-2105085-s001.pdf]

ADVANCED SCIENCE
Open Access

Supporting Information

for *Adv. Sci.*, DOI 10.1002/advs.202105085

Wide-Bandgap Organic–Inorganic Lead Halide Perovskite Solar Cells

Yao Tong, Adel Najar, Le Wang, Lu Liu, Minyong Du, Jing Yang, Jianxun Li, Kai Wang and Shengzhong (Frank) Liu**

Supporting information

Wide-bandgap Organic-inorganic Lead Halide Perovskite Solar Cells

Yao Tong, Adel Najar, Le Wang, Lu Liu, Minyong Du, Jing Yang, Jianxun Li, Kai Wang, Shengzhong (Frank) Liu**

Y. Tong and L. Wang
Faculty of Light Industry and Chemical Engineering, Dalian Polytechnic University, Dalian 116034, Liaoning, China
L. Liu, M. Du, J. Yang, J. Li, Dr. K. Wang and Prof. S. Z. Liu
Dalian National Laboratory for Clean Energy; iChEM, Dalian Institute of Chemical Physics, Chinese Academy of Sciences, Dalian 116023, Liaoning, China
Email: wangkai@dicp.ac.cn
Prof. S. Z. Liu
Key Laboratory of Applied Surface and Colloid Chemistry, Ministry of Education, Shaanxi Engineering Lab for Advanced Energy Technology, School of Materials Science and Engineering, Shaanxi Normal University, Xi'an 710119, Shaanxi, China
Email: szliu@dicp.ac.cn;
Prof. A. Najar
Department of Physics, College of Science, United Arab Emirates University, Al Ain 15505, United Arab Emirates.

Table S1. V_{OC} and PCE of PSCs based on WBG perovskites

Ref.	Perovskite	Eg[eV]	V_{OC}[V]	PCE[%]
[1]	$MAPb(I_{0.83}Br_{0.17})_3$	1.69	1.16	16.6
	$MAPb(I_{0.64}Br_{0.26})_3$	1.75	1.21	14.9
[2]	$FA_{0.83}Cs_{0.17}Pb(I_{0.6}Br_{0.4})_3$	1.74	1.20	17.1
[3]	$Cs_{0.2}FA_{0.8}Pb(I_{0.75}Br_{0.25})_3$	1.7	1.17	16.9
[3]	$Cs_{0.17}FA_{0.83}Pb(I_{0.6}Br_{0.4})_3$	1.78	1.21	15.7
[3]	$Cs_{0.12}MA_{0.05}FA_{0.83}Pb(I_{0.6}Br_{0.4})_3$	1.74	1.25	19.3
[4]	$Cs_{0.17}FA_{0.83}PbI_{1.5}Br_{1.5}$	1.86	1.296	15
[4]	$FA_{0.17}Cs_{0.83}PbI_{1.2}Br_{1.8}$	1.93	1.312	14.4
[4]	$Cs_{0.17}FA_{0.83}PbI_{1.8}Br_{1.2}$	1.79	1.284	16.5
[4]	$FA_{0.17}Cs_{0.83}PbI_{2.2}Br_{0.8}$	1.72	1.244	18.6
[5]	$MAPbI_{2.4}Br_{0.6}$	1.72	1.35	18.3
[6]	$Rb_{0.05}Cs_{0.095}MA_{0.1425}FA_{0.7125}PbI_2Br$	1.74	1.269	18.3

[7]	$FA_{0.83}Cs_{0.17}Pb(I_{0.6}Br_{0.4})_3$	1.75	1.31	19.8
[8]	$(FA_{0.83}MA_{0.17})_{0.95}Cs_{0.05}Pb(I_{0.6}Br_{0.4})_3$	1.71	1.20	18.3
[9]	$MAPbBr_3$	2.32	1.653	10.1
[9]	$MAPbBrI_2$	1.8	1.272	13.7
[10]	$FA_{0.6}Cs_{0.4}Pb(I_{0.65}Br_{0.35})_3$	1.81	1.22	16.3
[11]	$FAPbI_{1.5}Br_{1.5}/CsPbI_{1.5}Br_{1.5}$	1.82	1.1	17.1
[12]	$MA_{0.85}Cs_{0.15}Pb(I_{0.65}Br_{0.35})_3$	1.83	1.11	8.6
[13]	$Cs_{0.2}FA_{0.8}Pb(I_{0.6}Br_{0.4})_3$-DAP	1.84	1.26	15.2
[14]	$MAPb(I_{0.41}Br_{0.59})_3$	2.03	0.836	2.8
[15]	$MAPb(I_{0.3}Br_{0.7})_xCl_{3-x}(Br)$	2.04	1.34	10.3
[16]	$MAPbIBr_2$	2.05	1.45	6.1
[18]	$FAPbBr_3$	2.27	1.552	10.6
[19]	$MAPb_{0.75}Sn_{0.25}(I_{0.4}Br_{0.6})_3$	1.73	1.04	12.6
[19]	$MAPb_{0.75}Sn_{0.25}(I_{0.3}Br_{0.7})_3$	1.80	1.05	6.3
[20]	$FA_{0.8}Cs_{0.2}Pb(I_{0.7}Br_{0.3})_3$	1.75	1.23	17.7
[21]	$FA_{0.75}MA_{0.15}Cs_{0.1}Rb_{0.05}Pb(I_{0.67}Br_{0.33})_3$	1.73	1.13	17.3
[22]	$FA_{0.83}MA_{0.17}Pb(I_{0.6}Br_{0.4})_3$	1.72	1.15	17.2
[23]	$FA_{0.83}Cs_{0.17}Pb(I_{0.67}Br_{0.34})_3$	1.75	1.12	12.0
[24]	$FA_{0.15}Cs_{0.85}Pb(I_{0.73}Br_{0.27})_3$	1.72	1.24	18.1
[25]	$BA_{0.09}(FA_{0.83}Cs_{0.17})_{0.91}Pb(I_{0.6}Br_{0.4})_3$	1.72	1.18	17.2
[25]	$BA_{0.05}(FA_{0.83}Cs_{0.17})_{0.95}Pb(I_{0.8}Br_{0.2})_3$	1.61	1.14	20.6
[26]	$MAPb(I_{0.8}Br_{0.2})_3$	1.72	1.12	15.9
[26]	$MAPb(I_{0.5}Br_{0.5})_3$	1.87	1.21	10.6
[27]	$FA_{0.6}Cs_{0.4}Pb(I_{0.7}Br_{0.3})_3$	1.68	1.10	17.4
	$FA_{0.75}Cs_{0.25}Pb(I_{0.8}Br_{0.2})_3$	1.75	1.17	16.3
[28]	$FA_{0.8}Cs_{0.2}Pb(I0.6Br_{0.4})_3$	1.75	1.23	17.8
[29]	$MAPb(I_{0.67}Br_{0.33})_3$	1.77	1.08	10.7
[30]	$FA_{0.83}Cs_{0.17}Pb(I_{0.5}Br_{0.5})_3$	1.80	1.12	9.8
[31]	$MA_{0.90}Cs_{0.10}Pb(I_{0.6}Br_{0.4})_3$	1.82	1.22	12.5
[32]	$BAI_{0.8}Br_{0.2}$-$Cs_{0.6}MA_{0.4}Pb(I_{0.8}Br_{0.2})_3$	1.79	1.04	-
	$MA_{0.6}FA_{0.4}Pb(I_{0.6}Br_{0.4})_3$	1.76	1.17	14.7
[33]	$Cs_{0.6}FA_{0.4}Pb(I_{0.6}Br_{0.4})_3$	1.78	1.12	12.1
	$Cs_{0.6}MA_{0.4}Pb(I_{0.6}Br_{0.4})_3$	1.81	1.17	11.1
[34]	$Cs_{0.06}FA_{0.79}MA_{0.15}Pb(I_{0.4}Br_{0.6})_3$	1.78	1.23	17.5
[35]	$MAPb(I_{0.6}Br_{0.4})_3$	1.82	1.30	11.9
[36]	$MAPbI_{3-x}Cl_x$	1.55	1.13	19.3
[37]	BA-$MAPbI_3$	1.57	1.11	19.56
[38]	$MAPbI_3$	1.59	1.17	19.89
[39]	$Cs_{0.1}FA_{0.74}MA_{0.13}PbI_{2.48}Br_{0.39}$	1.62	1.14	20.08
[40]	$(FA_{0.95}PbI_{2.95})_{0.85}(MAPbBr_3)_{0.15}$	1.62	1.17	20.9
[41]	$FA_xMA_{1-x}PbBr_yI_{1-y}$	1.64	1.14	20.7
[41]	$FA_xMA_{1-x}PbBr_yI_{1-y}$	1.66	1.10	13.2
[41]	$FA_xMA_{1-x}PbBr_yI_{1-y}$	1.68	1.18	14.7
[41]	$FA_xMA_{1-x}PbBr_yI_{1-y}$	1.68	1.15	18.5
[42]	$MAPb(I_{0.8}Br_{0.2})_3$	1.72	1.02	13.1
[43]	$FA_{0.83}Cs_{0.17}Pb(I_{0.6}Br_{0.4})_3$	1.74	1.2	17.1
[44]	$Cs_x(MA_{0.17}FA_{0.83})_{(100-x)}Pb(I_{0.83}Br_{0.17})_3$	1.6	1.23	N.A.
[45]	(Rb/Cs/MA/FA)PbIBr	1.63	1.18	21.8
[46]	$DMA_{0.1}FA_{0.6}Cs_{0.3}PbI_{2.4}Br_{0.6}$	1.7	1.2	19.2
[47]	$MA_{0.9}Cs_{0.1}Pb(I_{0.6}Br_{0.4})_3$	1.8		15.1
[48]	$FAPbBr_3$	2.23	1.02	1.9
[49]	$FAPbBr_3$	2.23	1.35	6.5
[50]	$MAPbBr_3$-Cl	2.3	1.50	2.7
[51]	$MAPbI_{2.1}Br_{0.9}$	1.75	1.01	12.67

[52]	$MAPbBr_3$	2.3	1.45	8.7
[53]	$MAPbBr_3$	2.3	1.42	8.29
[54]	$MAPbBr_3$	2.3	1.61	7.5
[55]	$MAPbBr_3$	2.3	1.57	8.7
[56]	$MAPbBr_3$	2.3	1.51	10.4
[57]	$MAPbI_2Br$	1.8	1.04	11.03
[58]	$MAPbI_2Br$	1.8	1.09	10.03
[59]	$MAPbBr_3$	2.25	1.3	8.4
[60]	$FAPbBr_3$	2.26	1.53	8.2
[61]	$Cs_{0.15}(FA_{0.83}MA_{0.17})_{0.85}Pb(I_{0.7}Br_{0.3})_3$	1.70	1.19	18.6
[62]	$FA_{0.8}Cs_{0.2}PbI_{0.7}Br_{0.3})_3$	1.75	1.143	14
[63]	$Cs_{0.05}Rb_{0.05}FA_{0.765}MA_{0.135}PbI_{2.55}Br_{0.45}$	1.75	1.19	16.3
[64]	$MAPbBr_3$	2.3	1.47	8.9
[65]	$MAPbBr_3$	2.3	1.41	5.2
[66]	$MAPb(I_{0.83}Br_{0.27})_3$	1.71	1.24	16.74
[67]	$FA_{0.8}Cs_{0.2}PbI_{0.7}Br_{0.3})_3$	1.75	1.20	15.7
[68]	$Cs_{0.15}FA_{0.85}Pb(I_{0.3}Br_{0.7})_3$	2.00	1.2	10.7
[69]	$(FA_{0.58}GA_{0.10}Cs_{0.32})Pb(I_{0.73}Br_{0.27})_3$	1.75	1.24	14.2
[70]	$FA_{0.8}Cs_{0.2}Pb(I_{0.7}Br_{0.3})_3$	1.75	1.24	18.19
[71]	$FA_{0.8}Cs_{0.2}Pb(I_{0.3}Br_{0.7})_3$	2.0	1.18	11.5
[72]	$MA_{0.9}FA_{0.1}Pb(I_{0.6}Br_{0.4})_3$	1.81	1.21	17.1
[73]	$FA_{0.8}Cs_{0.2}Pb(I_{0.7}Br_{0.3})_3$	1.73	1.25	19.07
[74]	$(FA_{0.6}MA_{0.4})_{0.9}Cs_{0.1}Pb(I_{0.6}Br_{0.4})_3$	1.75	1.26	18.3
[75]	$FA_{0.8}Cs_{0.2}Pb(I_{0.6}Br_{0.4})_3$	1.77	1.2	16.4

We summarized the data on the basis of ref [35] and [7].

Reference

[1] M. Hu, C. Bi, Y. Yuan, Y. Bai, J. Huang. *Adv. Sci.*, **2016**, 3, 1500301.

[2] D. P. Mcmeekin, G. Sadoughi, W. Rehman, G. E. Eperon, M. Saliba, M. T. Hörantner, A. Haghighirad, N. Sakai, L. Korte, B. Rech, M. B. Johnston, L. M. Herz, H. J. Snaith. **2016**, 351, 151.

[3] H. Tan, F. Che, M. Wei, Y. Zhao, M. I. Saidaminov, P. Todorović, D. Broberg, G. Walters, F. Tan, T. Zhuang, B. Sun, Z. Liang, H. Yuan, E. Fron, J. Kim, Z. Yang, O. Voznyy, M. Asta, E. H. Sargent. *Nat. Commun.*, **2018**, 9, 3100.

[4] Y. Zhou, Y.-H. Jia, H.-H. Fang, M. A. Loi, F.-Y. Xie, L. Gong, M.-C. Qin, X.-H. Lu, C.-P. Wong, N. Zhao. *Adv. Funct. Mater*. **2018**, 28, 1803130.

[5] Z. Liu, J. Siekmann, B. Klingebiel, U. Rau, T. Kirchartz. *Adv. Energy Mater.*, **2021**, 11, 2003386.

[6] T. Duong, H. Pham, T. C. Kho, P. Phang, K. C. Fong, D. Yan, Y. Yin, J. Peng, M. A. Mahmud, S. Gharibzadeh, B. A. Nejand, I. M. Hossain, M. R. Khan, N. Mozaffari, Y. Wu, H. Shen, J. Zheng, H. Mai, W. Liang, C. Samundsett, M. Stocks, K. Mcintosh, G. G. Andersson, U. Lemmer, B. S. Richards, U. W. Paetzold, A. Ho-Ballie, Y. Liu, D. Macdonald, A. Blakers, J. Wong-Leung, T. White, K. Weber, K. Catchpole. *Adv. Energy Mater.*, **2020**, 10, 1903553.

[7] S. Gharibzadeh, B. Abdollahi Nejand, M. Jakoby, T. Abzieher, D. Hauschild, S. Moghadamzadeh, J. A. Schwenzer, P. Brenner, R. Schmager, A. A. Haghighirad, L. Weinhardt, U. Lemmer, B. S. Richards, I. A. Howard, U. W. Paetzold. *Adv. Energy Mater.*, **2019**, 9, 1803699.

[8] Y. Lin, B. Chen, F. Zhao, X. Zheng, Y. Deng, Y. Shao, Y. Fang, Y. Bai, C. Wang, J. Huang. *Adv. Mater.*, **2017**, 29, 1700607.

[9] X. Hu, X.-F. Jiang, X. Xing, L. Nian, X. Liu, R. Huang, K. Wang, H.-L. Yip, G. Zhou. *Solar RRL*. **2018**, 2, 1800083.

[10] Z. Yang, Z. Yu, H. Wei, X. Xiao, Z. Ni, B. Chen, Y. Deng, S. N. Habisreutinger, X. Chen, K. Wang, J. Zhao, P. N. Rudd, J. J. Berry, M. C. Beard, J. Huang. *Nat. Commun.*, **2019**, 10, 4498.

[11] Y.-N. Zhang, B. Li, L. Fu, Y. Zou, Q. Li, L.-W. Yin. *Sol. Energ. Mater. Sol. C.*, **2019**, 194, 168.
[12] M.-J. Wu, C.-C. Kuo, L.-S. Jhuang, P.-H. Chen, Y.-F. Lai, F.-C. Chen. *Adv. Energy Mater.*, **2019**, 9, 1901863.
[13] W.-Q. Wu, Z. Yang, P. N. Rudd, Y. Shao, X. Dai, H. Wei, J. Zhao, Y. Fang, Q. Wang, Y. Liu, Y. Deng, X. Xiao, Y. Feng, J. Huang. **2019**, 5, eaav8925.
[14] S. A. Kulkarni, T. Baikie, P. P. Boix, N. Yantara, N. Mathews, S. Mhaisalkar. *J. Mater. Chem. A*. **2014**, 2, 9221.
[15] Q. Xue, G. Chen, M. Liu, J. Xiao, Z. Chen, Z. Hu, X.-F. Jiang, B. Zhang, F. Huang, W. Yang, H.-L. Yip, Y. Cao. *Adv. Energy Mater.*, **2016**, 6,
[16] Y. Zhao, A. M. Nardes, K. Zhu. *Faraday Discuss.*, **2014**, 176, 301.
[17] T. Bu, X. Liu, R. Chen, Z. Liu, K. Li, W. Li, Y. Peng, Z. Ku, F. Huang, Y.-B. Cheng, J. Zhong. *J. Mater. Chem. A*. **2018**, 6, 6319.
[18] Y. Zhang, Y. Liang, Y. Wang, F. Guo, L. Sun, D. Xu. *ACS Energy Lett.*, **2018**, 3, 1808.
[19] Z. Yang, A. Rajagopal, S. B. Jo, C.-C. Chueh, S. Williams, C.-C. Huang, J. K. Katahara, H. W. Hillhouse, A. K. Y. Jen. *Nano Lett.*, **2016**, 16, 7739.
[20] Y. Yu, C. Wang, C. R. Grice, N. Shrestha, D. Zhao, W. Liao, L. Guan, R. A. Awni, W. Meng, A. J. Cimaroli, K. Zhu, R. J. Ellingson, Y. Yan. *ACS Energy Lett.*, **2017**, 2, 1177.
[21] T. Duong, Y. Wu, H. Shen, J. Peng, X. Fu, D. Jacobs, E.-C. Wang, T. C. Kho, K. C. Fong, M. Stocks, E. Franklin, A. Blakers, N. Zin, K. Mcintosh, W. Li, Y.-B. Cheng, T. P. White, K. Weber, K. Catchpole. *Adv. Energy Mater.*, **2017**, 7, 1700228.
[22] X. Zheng, B. Chen, J. Dai, Y. Fang, Y. Bai, Y. Lin, H. Wei, X. Zeng, J. Huang. *Nat. Energy*. **2017**, 2, 17102.
[23] R. J. Stoddard, F. T. Eickemeyer, J. K. Katahara, H. W. Hillhouse. *The Journal of Physical Chemistry Letters*. **2017**, 8, 3289.
[24] Y. Zhou, F. Wang, Y. Cao, J.-P. Wang, H.-H. Fang, M. A. Loi, N. Zhao, C.-P. Wong. *Adv. Energy Mater.*, **2017**, 7, 1701048.
[25] Z. Wang, Q. Lin, F. P. Chmiel, N. Sakai, L. M. Herz, H. J. Snaith. *Nat. Energy*. **2017**, 2, 17135.
[26] G. Longo, C. Momblona, M.-G. La-Placa, L. Gil-Escrig, M. Sessolo, H. J. Bolink. *ACS Energy Lett.*, **2018**, 3, 214.
[27] K. A. Bush, K. Frohna, R. Prasanna, R. E. Beal, T. Leijtens, S. A. Swifter, M. D. Mcgehee. *ACS Energy Lett.*, **2018**, 3, 428.
[28] J. Kim, M. I. Saidaminov, H. Tan, Y. Zhao, Y. Kim, J. Choi, J. W. Jo, J. Fan, R. Quintero-Bermudez, Z. Yang, L. N. Quan, M. Wei, O. Voznyy, E. H. Sargent. *Adv. Mater.*, **2018**, 30, 1706275.
[29] Y. Zhou, M. Yang, O. S. Game, W. Wu, J. Kwun, M. A. Strauss, Y. Yan, J. Huang, K. Zhu, N. P. Padture. *ACS Appl. Mater. Interfaces*. **2016**, 8, 2232.
[30] G. E. Eperon, T. Leijtens, K. A. Bush, R. Prasanna, T. Green, J. T.-W. Wang, D. P. Mcmeekin, G. Volonakis, R. L. Milot, R. May, A. Palmstrom, D. J. Slotcavage, R. A. Belisle, J. B. Patel, E. S. Parrott, R. J. Sutton, W. Ma, F. Moghadam, B. Conings, A. Babayigit, H.-G. Boyen, S. Bent, F. Giustino, L. M. Herz, M. B. Johnston, M. D. Mcgehee, H. J. Snaith. *Science*. **2016**, 354, 861.
[31] A. Rajagopal, Z. Yang, S. B. Jo, I. L. Braly, P.-W. Liang, H. W. Hillhouse, A. K.-Y. Jen. *Adv. Mater.*, **2017**, 29, 1702140.
[32] Z. Xiao, L. Zhao, N. L. Tran, Y. L. Lin, S. H. Silver, R. A. Kerner, N. Yao, A. Kahn, G. D. Scholes, B. P. Rand. *Nano Lett.*, **2017**, 17, 6863.
[33] S. Chen, Y. Hou, H. Chen, X. Tang, S. Langner, N. Li, T. Stubhan, I. Levchuk, E. Gu, A. Osvet, C. J.

Brabec. *Adv. Energy Mater.*, **2018**, 8, 1701543.

[34] M. Abdi-Jalebi, Z. Andaji-Garmaroudi, S. Cacovich, C. Stavrakas, B. Philippe, J. M. Richter, M. Alsari, E. P. Booker, E. M. Hutter, A. J. Pearson, S. Lilliu, T. J. Savenije, H. Rensmo, G. Divitini, C. Ducati, R. H. Friend, S. D. Stranks. *Nature*. **2018**, 555, 497.

[35] A. Rajagopal, R. J. Stoddard, S. B. Jo, H. W. Hillhouse, A. K. Y. Jen. *Nano Lett.*, **2018**, 18, 3985.

[36] H. Zhou, Q. Chen, G. Li, S. Luo, T.-B. Song, H.-S. Duan, Z. Hong, J. You, Y. Liu, Y. Yang. *Science*. **2014**, 345, 542.

[37] Y. Lin, Y. Bai, Y. Fang, Z. Chen, S. Yang, X. Zheng, S. Tang, Y. Liu, J. Zhao, J. Huang. *The Journal of Physical Chemistry Letters*. **2018**, 9, 654.

[38] Y. Bai, S. Xiao, C. Hu, T. Zhang, X. Meng, H. Lin, Y. Yang, S. Yang. **2017**, 7, 1701038.

[39] K. T. Cho, G. Grancini, Y. Lee, E. Oveisi, J. Ryu, O. Almora, M. Tschumi, P. A. Schouwink, G. Seo, S. Heo, J. Park, J. Jang, S. Paek, G. Garcia-Belmonte, M. K. Nazeeruddin. *Energ. Environ. Sci.*, **2018**, 11, 952.

[40] D. Luo, W. Yang, Z. Wang, A. Sadhanala, Q. Hu, R. Su, R. Shivanna, G. F. Trindade, J. F. Watts, Z. Xu, T. Liu, K. Chen, F. Ye, P. Wu, L. Zhao, J. Wu, Y. Tu, Y. Zhang, X. Yang, W. Zhang, R. H. Friend, Q. Gong, H. J. Snaith, R. Zhu. *Science*. **2018**, 360, 1442.

[41] T. Jesper Jacobsson, J.-P. Correa-Baena, M. Pazoki, M. Saliba, K. Schenk, M. Grätzel, A. Hagfeldt. *Energ. Environ. Sci.*, **2016**, 9, 1706.

[42] C. Bi, Y. Yuan, Y. Fang, J. Huang. *Adv. Energy Mater.*, **2015**, 5, 1401616.

[43] D. P. Mcmeekin, G. Sadoughi, W. Rehman, G. E. Eperon, M. Saliba, M. T. Hoerantner, A. Haghighirad, N. Sakai, L. Korte, B. Rech, M. B. Johnston, L. M. Herz, H. J. Snaith. *Science*. **2016**, 351, 151.

[44] J.-P. Correa-Baena, W. Tress, K. Domanski, E. H. Anaraki, S.-H. Turren-Cruz, B. Roose, P. P. Boix, M. Grätzel, M. Saliba, A. Abate, A. Hagfeldt. *Energ. Environ. Sci.*, **2017**, 10, 1207.

[45] M. Saliba, T. Matsui, J. Y. Seo, K. Domanski, J. P. Correa-Baena, M. K. Nazeeruddin, S. M. Zakeeruddin, W. Tress, A. Abate, A. Hagfeldt, M. Grätzel. *Energ. Environ. Sci.*, **2016**, 9, 1989.

[46] A. F. Palmstrom, G. E. Eperon, T. Leijtens, R. Prasanna, S. N. Habisreutinger, W. Nemeth, E. A. Gaulding, S. P. Dunfield, M. Reese, S. Nanayakkara, T. Moot, J. Werner, J. Liu, B. To, S. T. Christensen, M. D. Mcgehee, M. F. a. M. Van Hest, J. M. Luther, J. J. Berry, D. T. Moore. *Joule*. **2019**, 3, 2193.

[47] Y.-M. Xie, C. Ma, X. Xu, M. Li, Y. Ma, J. Wang, H. T. Chandran, C.-S. Lee, S.-W. Tsang. *Nano Res.*, **2019**, 12, 1033.

[48] G. E. Eperon, S. D. Stranks, C. Menelaou, M. B. Johnston, L. M. Herz, H. J. Snaith. *Energ. Environ. Sci.*, **2014**, 7, 982.

[49] F. C. Hanusch, E. Wiesenmayer, E. Mankel, A. Binek, P. Angloher, C. Fraunhofer, N. Giesbrecht, J. M. Feckl, W. Jaegermann, D. Johrendt, T. Bein, P. Docampo. *J. Phy. Chem. Lett.*, **2014**, 5, 2791.

[50] E. Edri, S. Kirmayer, M. Kulbak, G. Hodes, D. Cahen. *J. Phy. Chem. Lett.*, **2014**, 5, 429.

[51] W. Zhu, C. Bao, F. Li, X. Zhou, J. Yang, T. Yu, Z. Zou. *Chem. Commun.*, **2016**, 52, 304.

[52] R. Sheng, A. Ho-Baillie, S. Huang, S. Chen, X. Wen, X. Hao, M. A. Green. *J. Phy. Chem. C*. **2015**, 119, 3545.

[53] X. Zheng, B. Chen, C. Wu, S. Priya. *Nano Energy*. **2015**, 17, 269.

[54] C.-G. Wu, C.-H. Chiang, S. H. Chang. *Nanoscale*. **2016**, 8, 4077.

[55] L. Yongqi, W. Yajuan, M. Cheng, W. Sen, W. Xinnan, X. Dongsheng, S. Licheng. *Adv. Energy Mater.*, **2018**, 8, 1701159.

[56] H. J. Hyuck, S. D. Ho, I. S. Hyuk. *Adv. Mater.*, **2014**, 26, 8179.

[57] K. Cao, J. Cui, H. Zhang, H. Li, J. Song, Y. Shen, Y. Cheng, M. Wang. *J. Mater. Chem. A*. **2015**, 3, 9116.

[58] Y. Zhao, K. Zhu. *J. Am. Chem. Soc.*, **2014**, 136, 12241.

[59] J. H. Heo, S. H. Im. *Advanced materials (Deerfield Beach, Fla.)*. **2016**, 28, 5121.

[60] N. Arora, M. I. Dar, M. Abdi-Jalebi, F. Giordano, N. Pellet, G. Jacopin, R. H. Friend, S. M. Zakeeruddin, M. Grätzel. *Nano Lett.*, **2016**, 16, 7155.

[61] B. Chen, Z. Yu, K. Liu, X. Zheng, Y. Liu, J. Shi, D. Spronk, P. N. Rudd, Z. Holman, J. Huang. *Joule*. **2019**, 3, 177.

[62] D. Zhao, C. Chen, C. Wang, M. M. Junda, Z. Song, C. R. Grice, Y. Yu, C. Li, B. Subedi, N. J. Podraza, X. Zhao, G. Fang, R.-G. Xiong, K. Zhu, Y. Yan. *Nat. Energy*. **2018**, 3, 1093.

[63] H. Shen, T. Duong, J. Peng, D. Jacobs, N. Wu, J. Gong, Y. Wu, S. K. Karuturi, X. Fu, K. Weber, X. Xiao, T. P. White, K. Catchpole. *Energ. Environ. Sci.*, **2018**, 11, 394.

[64] N. K. Noel, B. Wenger, S. N. Habisreutinger, J. B. Patel, T. Crothers, Z. Wang, R. J. Nicholas, M. B. Johnston, L. M. Herz, H. J. Snaith. *ACS Energy Lett.*, **2018**, 3, 1233.

[65] R. Sheng, M. T. Hörantner, Z. Wang, Y. Jiang, W. Zhang, A. Agosti, S. Huang, X. Hao, A. Ho-Baillie, M. Green, H. J. Snaith. *The Journal of Physical Chemistry C*. **2017**, 121, 27256.

[66] D. B. Khadka, Y. Shirai, M. Yanagida, T. Noda, K. Miyano. *ACS Appl. Mater. Interfaces*. **2018**, 10, 22074.

[67] D. Zhao, C. Wang, Z. Song, Y. Yu, C. Chen, X. Zhao, K. Zhu, Y. Yan. *ACS Energy Lett.*, **2018**, 3, 305.

[68] D. Forgács, L. Gil-Escrig, D. Pérez-Del-Rey, C. Momblona, J. Werner, B. Niesen, C. Ballif, M. Sessolo, H. J. Bolink. *Adv. Energy Mater.*, **2017**, 7, 1602121.

[69] R. J. Stoddard, A. Rajagopal, R. L. Palmer, I. L. Braly, A. K. Y. Jen, H. W. Hillhouse. *ACS Energy Lett.*, **2018**, 3, 1261.

[70] C. Chen, Z. Song, C. Xiao, D. Zhao, N. Shrestha, C. Li, G. Yang, F. Yao, X. Zheng, R. J. Ellingson, C.-S. Jiang, M. Al-Jassim, K. Zhu, G. Fang, Y. Yan. *Nano Energy*. **2019**, 61, 141.

[71] D. Forgács, D. Pérez-Del-Rey, J. Ávila, C. Momblona, L. Gil-Escrig, B. Dänekamp, M. Sessolo, H. J. Bolink. *J. Mater. Chem. A*. **2017**, 5, 3203.

[72] Y.-M. Xie, Z. Zeng, X. Xu, C. Ma, Y. Ma, M. Li, C.-S. Lee, S.-W. Tsang. *Small*. **2020**, 16, 1907226.

[73] C. Chen, Z. Song, C. Xiao, R. A. Awni, C. Yao, N. Shrestha, C. Li, S. S. Bista, Y. Zhang, L. Chen, R. J. Ellingson, C.-S. Jiang, M. Al-Jassim, G. Fang, Y. Yan. *ACS Energy Lett.*, **2020**, 5, 2560.

[74] Z. Li, J. Zhang, S. Wu, X. Deng, F. Li, D. Liu, C. C. Lee, F. Lin, D. Lei, C.-C. Chueh, Z. Zhu, A. K. Y. Jen. *Nano Energy*. **2020**, 78, 105377.

[75] K. Xiao, R. Lin, Q. Han, Y. Hou, Z. Qin, H. T. Nguyen, J. Wen, M. Wei, V. Yeddu, M. I. Saidaminov, Y. Gao, X. Luo, Y. Wang, H. Gao, C. Zhang, J. Xu, J. Zhu, E. H. Sargent, H. Tan. *Nat. Energy*. **2020**, 5, 870.
